# Database coverage and their use in systematic reviews regarding spinal manipulative therapy: an exploratory study

**DOI:** 10.1186/s12998-022-00468-8

**Published:** 2022-12-19

**Authors:** Martin Nørregård Eybye, Simon Dyrløv Madsen, Anders Nikolai Ørsted Schultz, Casper Glissmann Nim

**Affiliations:** 1 Department of Sports Science and Clinical Biomechanics, University of Southern Denmark, Odense, Denmark; 2 The Chiropractic Knowledge Hub, Odense, Denmark; 3 Research Unit, Department of Internal Medicine, University Hospital of Southern Denmark, Sønderborg, Denmark; 4 Department of Regional Health Research, University of Southern Denmark, Odense, Denmark; 5 Medical Research Unit, Spine Center of Southern Denmark, University Hospital of Southern Denmark, Middelfart, Denmark

**Keywords:** Systematic review, Search strategy, Spinal manipulative therapy, Randomized controlled trial

## Abstract

**Background:**

Systematic reviews (SRs) of randomized controlled trials (RCTs) are considered one of the most reliable study types. Through a systematic and thorough literature search, researchers aim to collect all research relevant to their purpose. The selection of databases can be challenging and depend on the topic of interest. The Cochrane Handbook suggests searching at least the following three databases: Cochrane Library, MEDLINE, and EMBASE. However, this is not always sufficient for reviews on the musculoskeletal field in general.

This study aimed to examine the frequency and choice of databases used by researchers in SRs of spinal manipulative therapy (SMT). Secondly, to analyze the RCTs included in the SRs to determine the optimal combination of databases needed to conduct efficient literature searches for SRs of SMT.

**Methods:**

SRs investigating the effect of SMT on any patient-reported outcome measure were identified through searches in PubMed and Epistemonikos (all entries till date of search February 25, 2022). For each SR, databases searched and included RCTs were collected. RCTs were searched individually in nine databases (Cochrane Library, MEDLINE/PubMed, EMBASE, Google Scholar, CINAHL, Web of Science, Index to Chiropractic Literature, PEDro, and AMED). Coverage rates were calculated using the number of retrieved RCTs by the database or combinations of databases divided by the total number of RCTs.

**Results:**

Eighty-five SRs published met the inclusion criteria, and 442 unique RCTs were retrieved. The most frequently searched database was MEDLINE/PubMed. Cochrane Library had the highest overall coverage rate and contained the third most unique RCTs. While a 100% retrieval was not possible, as 18 RCTs could not be retrieved in any of the nine databases, the combination of Cochrane Library, Google Scholar, and PEDro retrieved all possible RCTs with a combined coverage rate of 95.9%.

**Conclusions:**

For SRs on SMT, we recommend using the combination suggested by the Cochrane Handbook of Cochrane Library, MEDLINE/PubMed, Embase, and in addition, PEDro and Index to Chiropractic Literature. Google Scholar might be used additionally as a tool for searching gray literature and quality assurance.

## Background

Systematic reviews (SRs) of randomized controlled trials (RCTs) are widely accepted to be on top of the evidence hierarchy [1, 2]. They are cornerstones in evidence-based healthcare [3] and evidence-based research [4]. This comes to fruition by condensing all relevant and available evidence on a topic and drawing a general conclusion from a broader population by combining sample sizes and thereby reducing biases [5]. In order to collect all relevant studies, a comprehensive literature search must be conducted, and researchers are generally advised to search multiple databases and use additional methods such as citation tracking, contacting experts in the field, and searching gray literature [6–12]. As the Cochrane Handbook for Systematic Reviews of Interventions highlights, leaving out relevant evidence can lead to selection bias. Cochrane thereby recommends searching at least the following three databases: The Cochrane Central Register of Controlled Trials (CENTRAL), MEDLINE, and EMBASE [11]. However, these recommendations may not sufficiently cover all relevant aspects of the research question. Some types of research or research topics may only be found in specialty journals that are not indexed in all databases [13]. An example of such could be literature related to chiropractic and, more specifically, spinal manipulative therapy (SMT) [14]. SMT is a guideline-recommended conservative therapy used by various practitioners, including chiropractors, osteopaths, and physiotherapists worldwide, typically to treat low back pain, neck pain, and headache [15, 16]. Furthermore, the procedures and theoretical frameworks have developed quite substantially over the last century [17]. It is not unlikely that specific papers are only published in journals related to those professions and thereby only found in the corresponding database.

In contrast, searching too many databases has clear disadvantages, as the search strategy must be translated to fit different databases using different interfaces and search syntaxes, and the time spent screening more, likely irrelevant, titles and abstracts is not insignificant [18]. Which and how many databases are necessary to be searched and the added value of select databases has been the topic of many previous studies, and the main takeaway seems that it, as expected, heavily depends on the topic of interest [13, 14, 19–30]. No research has looked systematically at retrieving relevant SMT papers. However, in the broader field of musculoskeletal disorders, Aagaard et al. [31] found MEDLINE, EMBASE, and CENTRAL to be insufficient at identifying all effect studies based on achieving a combined coverage rate of 88.9%. In an attempt to make a more generalized recommendation across all biomedical fields, Bramer et al. [32] found that searches should include EMBASE, MEDLINE, Web of Science, and Google Scholar as minimum requirements.

The Preferred Reporting Items for Systematic reviews and Meta-Analysis (PRISMA) tool was developed in 2009 to standardizing reporting in SRs, ensuring transparency and minimizing biases [33]. PRISMA and the use of an information specialist have become imperative when conducting a high-quality SR [34–36].

Hence, conducting a SR on a specific intervention such as SMT is not without challenge, and the selection of databases has not yet been explored sufficiently. This study will examine the frequency and choice of databases used by researchers in SRs of SMT. Secondly, to analyze the RCTs included in the SRs to determine the optimal combination of databases to conduct efficient literature searches for SRs of SMT. Finally, to examine whether the year of publication or the use of an information specialist influenced the number of investigated databases and how the use of PRISMA has changed over time.

## Methods

The research protocol for this study was registered at the Open Science Framework (protocol: https://osf.io/6ezxn/?view_only=3a750c9d398e4afa895c5a5d53346aa4).

### Changes made to the protocol

To ensure feasibility of completion, we had to limit our approach to SRs in English, Danish, Norwegian, and Swedish, exclude SRs focusing on more general conservative approaches, and SRs not focusing on patient-reported outcome measures (PROMs). SRs focusing on adverse events, cost-effectiveness, and age groups below 18 years were also excluded. Additionally, we searched all databases that were used in more than 20% of the SRs instead of the five most common.

### Eligibility criteria

We included SRs investigating the effect of spinal manipulations on any spinal region (i.e., cervical, thoracic, or lumbar spine, and the sacroiliac-joint (SI)). The SRs had to include RCTs evaluating any PROM. Exclusion criteria were (a) not an SR, (b) SRs focusing on more general conservative approaches, (c) SRs not evaluating PROMs, (d) SRs of age groups below 18 years, (e) SRs focusing on cost-effectiveness, (f) SRs focusing on adverse events, and (g) lack of full list of databases searched. The title and abstract screening process was performed independently by two researchers (MNE and SDM). Conflicts in the screening process of the SRs were solved by CGN and MNE by discussion.

All references included in the SRs were collected and manually evaluated. Hence, references investigating the effect of spinal manipulations on any spinal region using any PROM were included. Other study types and RCTs, including age groups below 18 years, and unpublished papers, were excluded.

### Search strategy

SRs investigating the effect of SMT were retrieved from PubMed and Epistemonikos [37] for all entries (date of search February 25, 2022). For PubMed, the search term “Musculoskeletal manipulations” [MeSH] and the filter “systematic reviews” was applied. For Epistemonikos, a search by title or abstract using the search terms combined with the Boolean operators (musculoskeletal OR spinal*) AND (manipulation* OR adjust* OR chiropract*) and filtered for systematic reviews was performed. No restriction to the date of publication was applied.

### Data collection

All variables collected are shown in Table 1. The body part related to the treated disorder was categorized into “cervical + headache”, “thoracic”, “lumbar + SI-joint + coccyx”, “extremities”, “multiple sites”, and “not defined”. We extracted information on which databases and search platforms were used in the SRs. For simplicity, we label these “databases” onwards. All included RCTs were manually searched in the following databases: MEDLINE/PubMed (via PubMed), CENTRAL (via Cochrane Library), EMBASE (via Ovid), jointly through Web of Science Core Collection Indexes (Science Citation Index Expanded, Social Sciences Citation Index, Arts and Humanities Citation Index, Conference Proceedings Citation Index (Science + Social Sciences and Humanities), and Emerging Sources Citation Index), henceforth listed as Web of Science, and Google Scholar. When searching Google Scholar, we searched the titles in quotations and unchecked the inclusion of citations. These databases were chosen because it allowed us to investigate the databases recommended by the Cochrane Handbook for SRs of Interventions and the previously suggested databases by Bramer et al. [32]. Furthermore, we also searched all other databases used by more than 20% of the included SRs in our study. As PubMed includes all MEDLINE references [38], we treated them as one database to avoid misleading results.

**Table 1:** Variables collected in this study

Variables from systematic reviews	Variables from retrieved randomized controlled trials
Author(s)	Title
Title	First author
Number of databases searched	Year of publication
Names of databases searched	Journal
Region of body related to treated disorder	Digital object identifier
Year of publication	
The use of an information specialist	
The use of PRISMA	
Number of included RCTs	

*PRISMA* the Preferred Reporting Items for Systematic reviews and Meta-Analysis, *RCT* randomized controlled trial

The RCTs were initially searched by title, and if that yielded no result, further searches using author, year of publication, and digital object identifier (DOI) were performed. MNE performed all searches, and SDM independently searched a sample of 50 random RCTs. We calculated intraclass correlation coefficient (ICC) using the two-way mixed-effects model to secure consistency in our search approach [39]. An ICC < 0.9 would lead to further training and collaboration between the two data curators.

### Statistical analysis

The number and frequency of databases searched were described in absolute numbers, mean, median, and interquartile range (IQR). The correlation between the number of databases searched and the year of publication of the included SRs was performed using Spearman’s rank correlation coefficient. Use of an information specialist was reported as number and frequency. The correlation was calculated using Pearson’s correlation coefficient. Correlation between the year of publication and the use of PRISMA was also performed using Pearson’s correlation coefficient. In this analysis, we only included SRs published after 2009, the year PRISMA was published [33].

The contribution of RCTs from each database and the various combinations of databases and their combined contributions were described as absolute numbers, overall coverage, mean coverage per SR, median coverage per SR, and 100% coverage per SR. Coverage rates were calculated using the numbers of RCTs retrieved by the database(s) divided by the total number of included RCTs, presented as percentages. Although Google Scholar has a high recall rate, previous reports have highlighted issues with low precision in structured literature searches of Google Scholar. Hence, calculations were performed with and without Google Scholar [40, 41]. We tabulated the three best combinations across two, three, and four databases, both including and excluding Google Scholar. All statistical analyses were performed in RStudio (v. 4.1.3, RStudio v. 1.4) for Windows 10 using the Tidyverse packages [42].

## Results

**Fig. 1:**
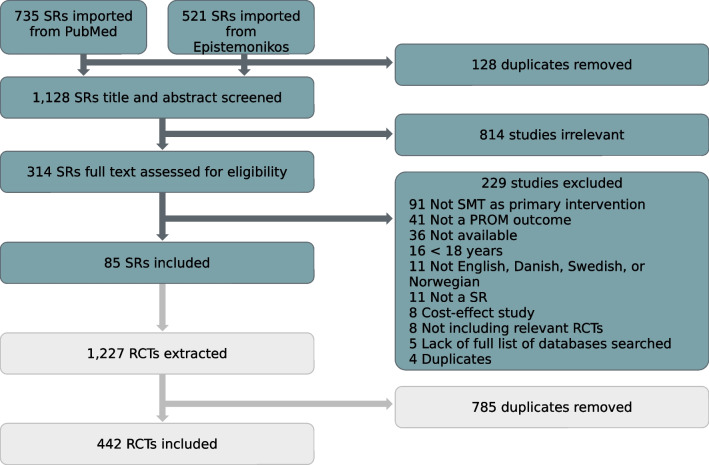
Flowchart of the selection process of the included and excluded SRs and RCTs

The initial searches in PubMed and Epistemonikos yielded 1,256 results, of which 128 were duplicates. After title and abstract screening, 314 SRs were eligible for full text review. Eighty-five SRs ended up being included. The 36 SRs where we could not access full-texts were excluded as this was an exploratory study, and resources were limited for acquiring additional materials. The full process can be seen in Fig. 1.

From the 85 included SRs, 1227 RCTs were collected, and after removing 785 duplicates, 442 (36%) unique titles were manually searched in the nine databases, the five previous stated and CINAHL (via EBSCOhost), Index to Chiropractic Literature (ICL) (Chiroindex.org – Index To Chiropractic Literature), PEDro (English – PEDro), and AMED – The Allied and Complementary Medicine Database (via EBSCOhost). MANTIS was also searched by more than 20% of the SRs, but despite multiple attempts, we could not gain access to MANTIS. From a sample of 50 random RCTs, an ICC of 0.97 (95% confidence interval = 0.96–0.97) showed excellent agreement between the two assessors without the need for further training.

### Characteristics of the included SRs and RCTs

**Fig. 2:**
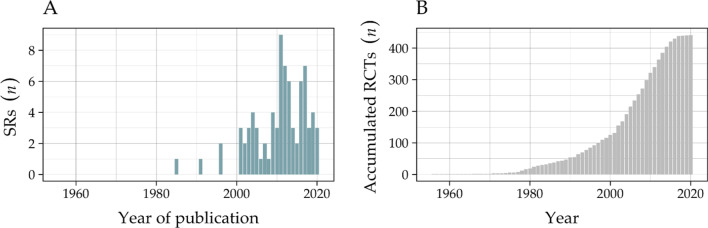
**A** Distribution of the included systematic reviews and **B** accumulation of included randomized controlled trials over time

All the included SRs were published between 1985 and 2021. Figure 2 shows the distribution over time for the included SRs and the RCTs’ accumulation. A significant but weak correlation of 0.25 was found, indicating that newer SRs search slightly more databases.

Thirty-four (40%) of the SRs investigated the effect of SMT as a treatment for disorders in the lumbar spine, SI-joint, or coccyx. The second most investigated region was the cervical spine and different types of headaches, which 25 (29%) of the SRs focused on.

Sixteen (19%) of the SRs included used an information specialist. No correlation was found between the use of an information specialist/research librarian and the number of databases searched.

**Fig. 3:**
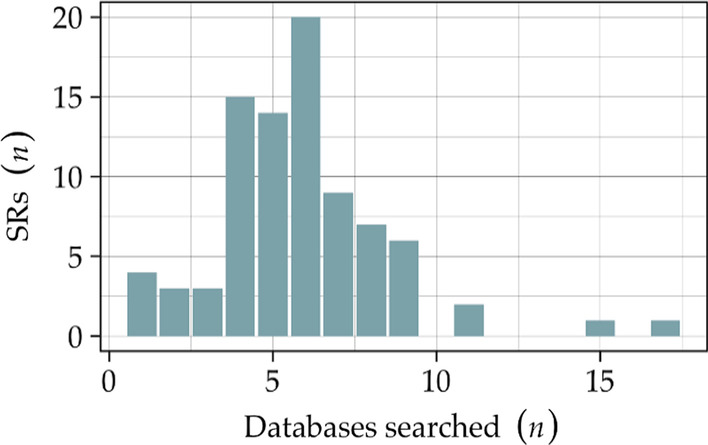
Number of databases searched by the systematic reviews

**Fig. 4:**
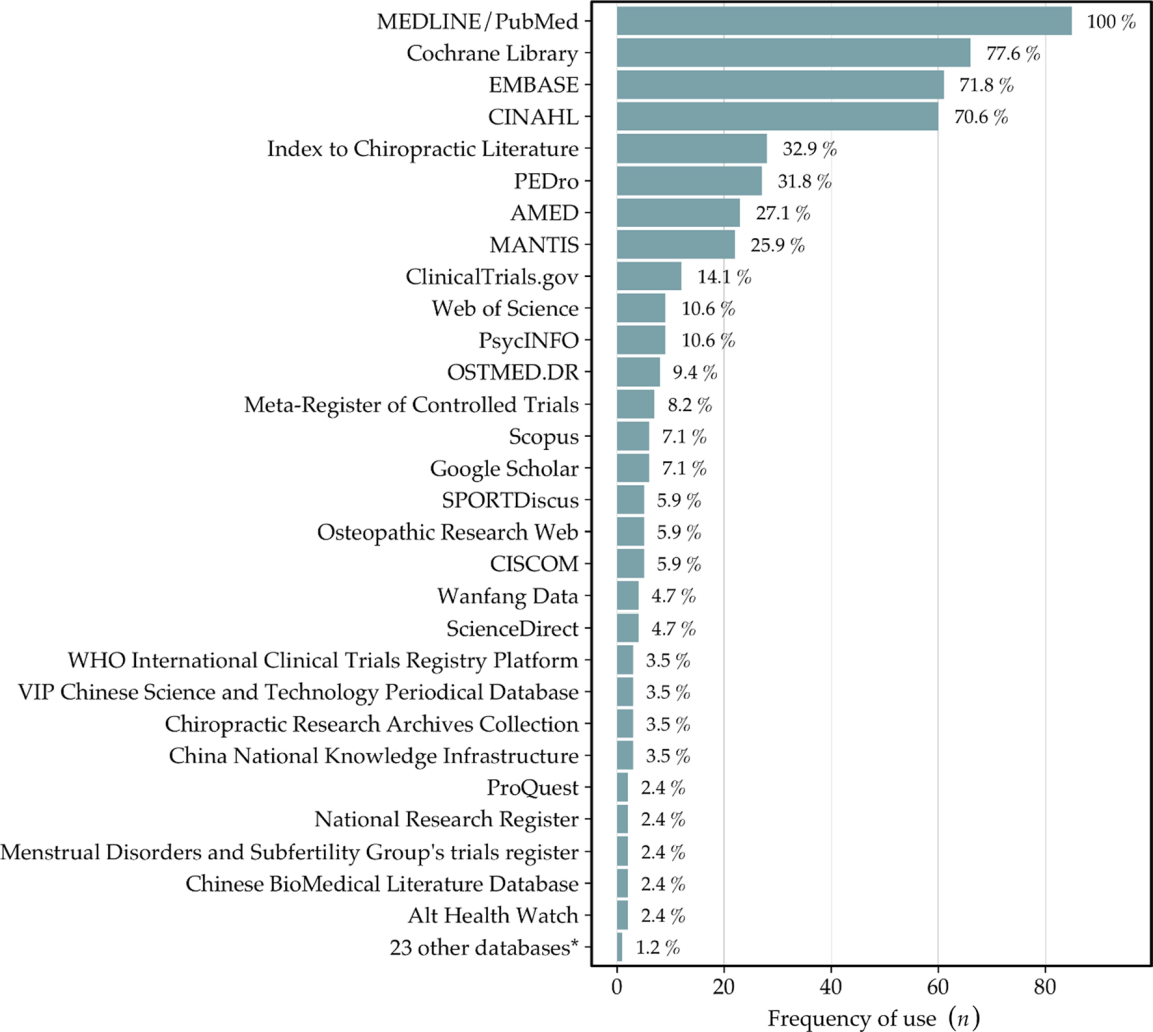
Frequency of use of individual databases by the included systematic reviews

Mean and median numbers for databases searched by the SRs were 5.8 and 6, respectively, with an IQR of 3, the distribution is shown in Fig. 3. All 85 SRs searched MEDLINE/PubMed (100%), Cochrane Library (78%), EMBASE (72%), and CINAHL (71%) were searched second to fourth most, with a considerable drop to the fifth most searched database being the Index to Chiropractic Literature at 33%. Collectively, the 85 SRs searched 52 different databases, shown in Fig. 4. Mean, median, and IQR for RCTs per SR were 14.4, 8, and 15, respectively. No correlation was found between the number of RCTs per SR, and the number of databases searched (correlation coefficient = − 0.06).

Fifty-eight SRs were published after 2009 and included in the correlation calculation between the use of PRISMA and the year of publication. Twenty-nine of the 58 SRs (50%) reported using PRISMA. A significant moderate correlation of 0.68 between the use of PRISMA and the year of publication was found, indicating that more recent SRs more often apply PRISMA.

Appendix 1 contains a full list of the included SRs and their characteristics. Appendix 2 provides a complete list of all included randomized controlled trials, their characteristics, and in which databases they were found.

### Unique RCTs per database

Eighteen of the 442 RCTs (4.1%) were not found in any of the nine databases. Thirteen (2.9%) RCTs were unique to only one database, Google Scholar (n = 6), PEDro (n = 4), and CENTRAL (n = 3). When excluding Google Scholar from the analysis 24 of the 442 RCTs (5.4%) were not found in any of the eight databases. Ten (2.3%) RCTs were unique to only one database, PEDro (n = 5), Cochrane Library (n = 4), and Index to Chiropractic Literature (n = 1), listed in Table 2. The 18 RCTs not found in any of the nine databases were primarily in Chinese, further details are listed in Table 3

**Table 2:** Number of unique RCTs per database

Databases	Unique RCTs in the database (*n*)	Unique RCTs in the databases—excl. Google Scholar (*n*)
PEDro	4	5
Cochrane Library	3	4
Index to Chiropractic Literature	0	1
MEDLINE/PubMed	0	0
EMBASE	0	0
Web of Science	0	0
CINAHL	0	0
AMED	0	0
Google Scholar	6	–

*RCT* randomized controlled trial, *SR* systematic review

**Table 3:** The 18 RCTs not found in any of the nine searched databases

Characteristics of non-retrievable RCTs	Number of RCTs
Chinese literature, including dissertations	11
German Osteopathic literature	5
Dissertations or master theses	2

*RCT* randomized controlled trial

### Coverage rates

For each of the databases, their overall coverage rate was calculated, and Cochrane Library obtained the highest individual coverage rate of 91.6%, followed by Google Scholar (88.2%) and EMBASE (85.5%).

Combined recall rates of three databases performed better, with the highest recall rate at 95.9% obtained by CENTRAL, Google Scholar, and PEDro. This combination was able to retrieve all 424 possible RCTs. The best combinations of four performed similarly, though the best performing combination of four databases excluding Google Scholar retrieved one more RCT (n = 418), than the best combination of three. The minimum recall per SR was zero for all nine databases due to one SR, including four RCTs, that were not found by any database. Tables 4, 5, 6 and 7 shows overall recall rates, mean, median, and 100% recall per SR of all individual databases and the three best performing combinations with and without Google Scholar. A complete list of all combinations of two, three and, four databases and their recall rates can be found in Appendix 3.

**Table 4:** Coverage rates of individual databases

Databases	RCTs found (*n*)	Overall recall^a^ (%)	Mean recall per SR^b^ (%)	Median recall per SR^c^ (%)	100% recall per SR^d^ (%)
CENTRAL	405	91.6	92.1	100.0	75.3
Google Scholar	390	88.2	88.7	95.2	47.1
EMBASE	378	85.5	87.6	100.0	50.6
PEDro	366	82.8	85.0	93.8	37.6
MEDLINE/PubMed	366	82.8	83.8	90.0	36.5
Web of Science	320	72.4	76.7	82.1	24.7
CINAHL	290	65.6	62.9	66.7	17.6
AMED	225	50.9	54.5	55.6	8.2
ICL	84	19.0	19.6	14.9	2.4
Total RCTs found by any of the nine databases investigated	424	95.9	–	–	–

*RCT* randomized controlled trial, *SR* systematic review

**Table 5:** Combined coverage rates of two databases

	RCTs found (*n*)	Overall recall^a^ (%)	Mean recall per SR^b^ (%)	Median recall per SR^c^ (%)	100% recall per SR^d^ (%)
*Combination of two databases incl. Google Scholar*					
Cochrane Library + Google Scholar	417	94.3	95.2	100.0	83.5
Google Scholar + PEDro	416	94.1	96.4	100.0	83.5
Cochrane Library + PEDro	414	93.7	94.3	100.0	82.4
*Combination of two databases excl. Google Scholar*					
Cochrane Library + PEDro	414	93.7	94.3	100.0	82.4
Cochrane Library + AMED	410	92.8	93.3	100.0	82.4
Cochrane Library + ICL	409	92.5	93.2	100.0	80.0

*RCT* randomized controlled trial, *SR* systematic review

^a^Overall recall: The total number of included references retrieved by the database(s) divided by the total number of included references

^b^Mean recall per SR: The average recall rate per SR

^c^Median recall per SR: The median value of recall per SR

^d^100% recall per SR: The percentage of SRs for which the database(s) retrieved all included references

**Table 6:** Combined coverage rates of three databases

	RCTs found (*n*)	Overall recall^a^ (%)	Mean recall per SR^b^ (%)	Median recall per SR^c^ (%)	100% recall per SR^d^ (%)
*Combination of three databases incl. Google Scholar*					
Cochrane Library + Google Scholar + PEDro	424	95.9	97.0	100.0	90.6
Cochrane Library + Google Scholar + AMED	420	95.0	95.6	100.0	88.2
Google Scholar + PEDro + AMED	419	94.8	96.8	100.0	85.9
*Combination of three databases excl. Google Scholar*					
Cochrane Library + EMBASE + PEDro	417	94.3	95.2	100.0	85.9
Cochrane Library + ICL + PEDro	417	94.3	95.4	100.0	85.9
Cochrane Library + MEDLINE/PubMed + PEDro	417	94.3	95.2	100.0	85.9

*RCT* randomized controlled trial, *SR* systematic review

^a^Overall recall: The total number of included references retrieved by the database(s) divided by the total number of included references

^b^Mean recall per SR: The average recall rate per SR

^c^Median recall per SR: The median value of recall per SR

^d^100% recall per SR: The percentage of SRs for which the database(s) retrieved all included references

**Table 7:** Combined recall rates of four databases

	RCTs found (*n*)	Overall recall^a^ (%)	Mean recall per SR^b^ (%)	Median recall per SR^c^ (%)	100% recall per SR^d^ (%)
*Combination of four databases incl. Google Scholar*					
Cochrane Library + Google Scholar + PEDro + CINAHL	424	95.9	97.0	100.0	90.6
Cochrane Library + Google Scholar + EMBASE + PEDro	424	95.9	97.0	100.0	90.6
Cochrane Library + Google Scholar + PEDro + ICL	424	95.9	97.0	100.0	90.6
*Combination of four databases incl. Google Scholar*					
Cochrane Library + CINAHL + PEDro + ICL	418	94.6	95.6	100.0	87.1
Cochrane Library + EMBASE + PEDro + ICL	418	94.6	95.6	100.0	87.1
Cochrane Library + MEDLINE/PubMed + PEDro + ICL	418	94.6	95.6	100.0	87.1

*RCT* randomized controlled trial, *SR* systematic review

^a^Overall recall: The total number of included references retrieved by the database(s) divided by the total number of included references

^b^Mean recall per SR: The average recall rate per SR

^c^Median recall per SR: The median value of recall per SR

^d^100% recall per SR: The percentage of SRs for which the database(s) retrieved all included references

## Discussion

On average, the SRs searched 5.8 databases, commonly corresponding to the Cochrane Handbook for Systematic Reviews of Interventions (i.e., MEDLINE/PubMed, Cochrane Library, and EMBASE) [11]. The SRs contained 14.4 RCTs on average, with an IQR of 15, indicating a large variation in research available depending on the topic within SMT. The large proportion of duplicate RCTs (64%) within all the included SRs, reflect a considerable overlap with many similar SRs on SMT in general.

The single database with the highest overall coverage rate was Cochrane Library (91.6%). It also outperformed the other databases on mean, median, and 100% coverage per SR, retrieving all RCTs in 75.3% of the included SRs. Adding Google Scholar, the coverage rate increased to 94.3%, only seven short of detecting the 424 possible RCTs. Excluding Google Scholar, the combination of Cochrane Library and PEDro retrieved 93.7% of all RCTs. The best combination of three databases, Cochrane Library, Google Scholar, and PEDro, was able to retrieve all possible RCTs with a coverage rate of 95.9%. When excluding Google Scholar, the best combination was Cochrane Library, PEDro, and ICL or EMBASE, with a coverage rate of 94.3%, retrieving eight more RCTs than Cochrane Library, MEDLINE/PubMed, and EMBASE combined, as recommended by the Cochrane Handbook. Although CINAHL was used more frequently than PEDro and performed better on its own than ICL, we suggest using PEDro or ICL over CINAHL when searching multiple databases. This is mainly due to fact that PEDro and ICL performed better than CINAHL when combined with Cochrane Library or both Cochrane Library and MEDLINE/PubMed. Furthermore, CINAHL did not retrieve any unique RCTs, while PEDro and ICL retrieved five and one unique RCTs, respectively, when excluding Google Scholar from the analysis.

Bramer et al. [32] suggested that an acceptable literature search for a SR should cover at least 95% of all possible studies. This was possible using any combination of Cochrane Library, Google Scholar, and PEDro/EMBASE/ICL. However, 18 RCTs were not found in any of the nine databases investigated in this study, resulting in the highest possible coverage rate being 95.9% (94.6% when excluding Google Scholar). However, we still find our results representative for conducting a thorough search as the same 18 RCTs limited our findings. Two of the included SRs contained 12 of the 18 RCTs not found, and for these apply, that they searched in either Chinese databases or databases explicitly related to osteopathy. The rest were found in six different SRs. The major challenge was Chinese literature (n = 11). Most likely because they are only indexed in databases other than the ones we searched, although issues relating to translation cannot be ruled out. The large diversity in databases searched by the SRs, especially Asian databases, and the amount of Chinese studies not found might suggest that a wide diversity of electronic databases is required to find all relevant materials. Our findings underline this, where PEDro found most unique references when ignoring Google scholar. Further research should aim to determine the role of Asian databases when performing SRs of SMT. Moreover, authors should remember the importance of a wide diversity of electronic databases combined with additional methods than electronic databases when searching for literature. These methods include hand searching journals, conference proceedings, searching reference lists of previously conducted systematic and narrative reviews, contacting experts in the field, and searching databases related to theses and dissertations [11, 12]. Searching ongoing and unpublished studies (often referred to as gray literature) also make up an important part of a systematic literature search, but since unpublished literature was excluded from this study, we cannot provide any specific considerations.

Overall, our results suggest that, in theory, using Cochrane's recommended databases along with PEDro and ICL appears sufficient to capture more than 95% of all SMT RCTs. Supporting the results of Aagaard et al. [31], who concluded that searching MEDLINE, EMBASE, and CENTRAL were insufficient when searching for musculoskeletal disorders. In their study, adding PEDro or ICL did not improve their search. However, their scope was much broader than ours. It is not unlikely that our findings can be extrapolated into manual therapy in general, as different types of interventions typically share (1) journals, (2) keywords, and (3) professions who administrate them. However, this is entirely speculative. Given our findings, we suggest that when performing reviews related to specific professions (e.g., chiropractic), selecting profession-specific databases (e.g., PEDro or ICL) in addition to Cochrane's recommended databases may provide more unique RCTs. Likewise, a review with another profession-oriented approach than chiropractic or physiotherapy could arguably exchange the ICL/PEDro for another profession-related database (e.g., Osteopathic Research Web for osteopaths).

Ranking databases based on coverage rates presents some challenges. The presence of a relevant study in a database does not automatically correspond to that study being found by the search strategy used (e.g., the selected keywords). This limitation becomes evident in the case of Google Scholar. Google Scholar achieved the second-highest single database coverage rate of 88.2% and was a part of the combinations with the highest coverage rates. Also, Google Scholar was able to identify the most unique RCTs (six) of all databases. Despite these impressive results, it has previously been reported that the precision of Google Scholar is low [40, 41]. Because of this and other limitations in its search functions, it has been assessed to be inadequate as a standalone database and should rather be used in addition to traditional databases [43]. An example could be to search for gray literature and quality assurance.

Only 16 (19%) of the SRs reported the use of an information specialist contradicting general guidelines [11] and suggestions from previous studies [34, 35]. However, this may be the result of under-reporting [35]. We highly suggest using an information specialist when conducting a SR since it enhances the quality of the SR [35, 44]. We would also remind researchers to report the use of information specialists when used, and acknowledge their work, either as an author, if they qualify for that according to the Vancouver guidelines, or in acknowledgments [45].

The increased number of databases searched and the increased use of PRISMA in recent years may reflect a tendency towards more emphasis on thorough methodology and transparency. This may explain the large number of duplicates found. Earlier SRs may be of such low quality that the information disserted is inapplicable to clinical practice, and newer SRs provide a more thorough and detailed dissertation. While this is speculative, some evidence suggests that the manual therapy professions have provided higher quality research in recent years [46, 47].

### Limitations

The assumption that the 442 included RCTs make up all relevant effect studies in the field of SMT is idealistic, as we did not perform a thorough systematic search and data extraction but an exploratory study. First, other SRs may have been found in databases other than PubMed and Epistemonikos or without using the “Systematic review” filter. Second, the included SRs may have excluded RCTs considered irrelevant for their purpose but could have been relevant in the context of contribution of databases. Third, the included SRs were published from 1985 to 2021 and may not include most recent RCTs. Fourth, older SRs may not have had access to the same databases as today. An example of this is Google Scholar, which was first released in 2004 [48], prior SRs would not have been able to use that database. However, despite all of this, we consider our sample size sufficient to provide thorough recommendations for future SMT reviews.

As mentioned above, evaluating databases solely on their performance in coverage rate and ability to find unique RCTs alone is not adequate. The fact that a database contains a reference is not the same as that reference being found using a search string or that a link to the full text is available. Our findings cannot be directly generalized to other fields as the performance of the databases greatly depends on the topic. Another limitation revolves around the selection of investigated databases. Our findings might look different if additional profession-specific databases were included (e.g., those related to osteopathy).

## Conclusion

Cochrane Library had the single highest overall coverage rate and contained the third most unique RCTs of the nine databases investigated. The combination which performed best excluding Google Scholar, was Cochrane Library, PEDro, Index to Chiropractic Literature and either EMBASE, MEDLINE/PubMed, or CINAHL, with a coverage of 94.6%.

For studies related to SMT, we suggest following the recommendations by the Cochrane Handbook searching Cochrane Library, MEDLINE, and EMBASE and adding PEDro and Index to Chiropractic Literature. In addition, Google Scholar might also be used to search gray literature and quality assurance or can be included in the search strategy depending on authors’ available research time and ambition.

Researchers should apply these results to select the most relevant databases for future SMT reviews. Furthermore, our findings should be translated to other areas of manual therapy.

## Data Availability

All data is available in the supplementary material. For details on the coding procedudres please contact casper.nim@rsyd.dk.

## Appendix 1

See Table 8.

**Table 8:** Overview of the included SRs

Title	Author(s)	Year of publication	Journal	Number of databases searched	Number of included RCTs	Number of included RCTs not found by any of the nine databases
Manipulation and mobilization of the cervical spine. A systematic review of the literature	Hurwitz et al.	1996	Spine	4	15	0
Efficacy of Spinal Manipulation for Chronic Headache: A Systematic Review	Bronfort et al.	2001	Journal of Manipulative and Physiological Therapeutics	5	10	0
Spinal manipulation for primary and secondary dysmenorrhoea	Proctor et al.	2001	Cochrane Database of Systematic Reviews	11	5	0
Spinal manipulation: a systematic review of sham-controlled, double-blind, randomized clinical trials Sham-Controlled, Double-Blind, Randomized Clinical Trials	Ernst et al.	2001	Journal of Pain and Symptom Management	5	6	0
High-velocity low-amplitude spinal manipulation for symptomatic lumbar disk disease: a systematic review of the literature	Lisi et al.	2005	Journal of Manipulative and Physiological Therapeutics	4	1	0
The effect of thoracic spine manipulation on pain and disability in patients with non-specific neck pain: a systematic review	Huisman et al.	2013	Disability and Rehabilitation	4	10	0
Spinal manipulative therapy for acute low back pain: an update of the cochrane review	Rubinstein et al.	2013	Spine	6	20	0
Osteopathic manipulative treatment for low back pain: a systematic review and meta-analysis of randomized controlled trials	Licciardone et al.	2005	BMC Musculoskeletal Disorders	9	6	0
Clinical effectiveness of the activator adjusting instrument in the management of musculoskeletal disorders: a systematic review of the literature	Huggins et al.	2012	Journal of the Canadian Chiropractic Association	5	5	0
Chiropractic care for nonmusculoskeletal conditions: a systematic review with implications for whole systems research	Hawk et al.	2007	Journal of Alternative and Complementary Medicine	5	4	0
Osteopathic intervention in chronic non-specific low back pain: a systematic review	Orrock et al.	2013	BMC Musculoskeletal Disorders	9	2	0
Spinal manipulation for asthma: a systematic review of randomised clinical trials	Ernst E	2009	Respiratory Medicine	4	1	0
Thoracic manual therapy is not more effective than placebo thoracic manual therapy in patients with shoulder dysfunctions: A systematic review with meta-analysis	Bizzarri et al.	2018	Musculoskeletal Science and Practice	7	5	0
Osteopathic manipulative treatment for low back and pelvic girdle pain during and after pregnancy: A systematic review and meta-analysis	Franke et al.	2017	Journal of Bodywork and Movement Therapies	8	3	0
Comparison Between Oblique Pulling Spinal Manipulation and Other Treatments for Lumbar Disc Herniation: A Systematic Review and Meta-Analysis	Mo et al.	2018	Journal of Manipulative and Physiological Therapeutics	8	9	8
Benefits and harms of spinal manipulative therapy for the treatment of chronic low back pain: systematic review and meta-analysis of randomised controlled trials	Rubinstein et al.	2019	BMJ	6	47	1
The effectiveness of thoracic spine manipulation for the management of musculoskeletal conditions: a systematic review and meta-analysis of randomized clinical trials	Walser et al..	2009	Journal of Manual and Manipulative Therapy	5	13	0
Osteopathic manipulative treatment: A systematic review and critical appraisal of comparative effectiveness and health economics research	Steel et al..	2017	Musculoskeletal Science and Practice	6	6	0
Is manipulative therapy clinically necessary for relief of neck pain? A systematic review and meta-analysis	Yao et al..	2017	Chinese Journal of Integrative Medicine	17	19	1
Effectiveness and Economic Evaluation of Chiropractic Care for the Treatment of Low Back Pain: A Systematic Review of Pragmatic Studies	Blanchette et al..	2016	PLoS One	5	8	0
Osteopathic manipulative treatment in neurological diseases: Systematic review of the literature	Cerritelli et al..	2016	Journal of the Neurological Sciences	11	4	0
Chinese manipulation for mechanical neck pain: a systematic review	Lin et al..	2012	Clinical Rehabilitation	4	4	0
The effectiveness of sub-group specific manual therapy for low back pain: a systematic review	Slater et al..	2012	Manual Therapy	4	7	0
The effectiveness of acupuncture, acupressure and chiropractic interventions on treatment of chronic nonspecific low back pain in Iran: A systematic review and meta-analysis	Yeganeh et al..	2017	Complementary Therapies in Clinical Practice	7	3	0
Association of Spinal Manipulative Therapy With Clinical Benefit and Harm for Acute Low Back Pain: Systematic Review and Meta-analysis	Paige et al..	2017	JAMA	4	28	0
Manipulation and mobilisation for neck pain contrasted against an inactive control or another active treatment	Gross et al.	2015	Cochrane Database of Systematic Reviews	7	62	0
Vertigo and Balance Disorders—The Role of Osteopathic Manipulative Treatment: A Systematic Review	Tramontano et al.	2021	Complementary Medicine Research	3	2	0
Spinal manipulative therapy in the management of cervicogenic headache	Fernández-de-Las-Peñas et al.	2005	Headache	6	2	0
The outcomes of manipulation or mobilization therapy compared with physical therapy or exercise for neck pain: a systematic review	Schroeder et al.	2013	Evidence-Based Spine-Care Journal	2	7	0
Osteopathy for musculoskeletal pain patients: a systematic review of randomized controlled trials	Posadzki et al.	2011	Clinical Rheumatology	6	14	0
Osteopathic manipulative treatment for nonspecific low back pain: a systematic review and meta-analysis	Franke et al.	2014	BMC Musculoskeletal Disorders	8	10	1
Patient-centered outcomes of high-velocity, low-amplitude spinal manipulation for low back pain: a systematic review	Goertz et al.	2012	Journal of Electromyography and Kinesiology	3	38	0
A Systematic Review and Meta-Analysis of the Efficacy of Manipulative Therapy in Women with Primary Dysmenorrhea	Abaraogu et al.	2017	Explore	6	3	0
Effectiveness of osteopathic manipulative therapy for managing symptoms of irritable bowel syndrome: a systematic review	Müller et al.	2014	The Journal of the American Osteopathic Association	6	2	0
The global summit on the efficacy and effectiveness of spinal manipulative therapy for the prevention and treatment of non-musculoskeletal disorders: a systematic review of the literature	Côté et al.	2021	Chiropractic and Manual Therapies	5	7	0
Determining the level of evidence for the effectiveness of spinal manipulation in upper limb pain: A systematic review and meta-analysis	Aoyagi et al.	2015	Manual Therapy	15	6	1
Are manual therapies, passive physical modalities, or acupuncture effective for the management of patients with whiplash-associated disorders or neck pain and associated disorders? An update of the Bone and Joint Decade Task Force on Neck Pain and Its Associated Disorders by the OPTIMa collaboration	Wong et al.	2016	The Spine Journal	5	9	0
Is manipulative therapy more effective than sham manipulation in adults: a systematic review and meta-analysis	Scholten-Peeters et al.	2013	Chiropractic and Manual Therapies	5	16	0
Effects of spinal manipulation versus therapeutic exercise on adults with chronic low back pain: a literature review	Merepeza A	2014	Journal of the Canadian Chiropractic Association	4	3	0
Spinal manipulation and mobilisation for back and neck pain: a blinded review	Koes et al.	1991	BMJ	1	35	0
Mobilization and Manipulation of the Cervical Spine in Patients with Cervicogenic Headache: Any Scientific Evidence?	Garcia et al.	2016	Frontiers in Neurology	6	9	0
Spinal manipulative therapy for low back pain. A meta-analysis of effectiveness relative to other therapies	Assendelft et al.	2003	Annals of Internal Medicine	4	53	0
Spinal manipulative therapy for acute low‐back pain	Rubinstein et al.	2012	Cochrane Database of Systematic Reviews	8	30	0
The effectiveness of physiotherapy and manipulation in patients with tension-type headache: a systematic review	Lenssinck et al.	2004	Pain	3	4	0
Effectiveness of physical therapist administered spinal manipulation for the treatment of low back pain: a systematic review of the literature	Kuczynski et al.	2012	International Journal of Sports Physical Therapy	6	6	0
Combined chiropractic interventions for low‐back pain	Walker et al.	2010	Cochrane Database of Systematic Reviews	6	19	0
Chiropractic Care of Adults With Postpartum-Related Low Back, Pelvic Girdle, or Combination Pain: A Systematic Review	Weis et al.	2020	Journal of Manipulative and Physiological Therapeutics	4	3	0
Manipulation and mobilization for treating chronic low back pain: a systematic review and meta-analysis	Coulter et al.	2018	The Spine Journal	6	64	0
Comparative effectiveness of exercise, acupuncture, and spinal manipulation for low back pain	Standaert et al.	2011	Spine	2	2	0
Spinal manipulations for cervicogenic headaches: a systematic review of randomized clinical trials	Posadzki et al.	2011	Headache	7	8	0
Osteopathic manipulative treatment (OMT) for lower urinary tract symptoms (LUTS) in women	Franke et al.	2013	Journal of Bodywork and Movement Therapies	9	4	4
The effectiveness of spinal manipulation for the treatment of headache disorders: a systematic review of randomized clinical trials	Astin et al.	2002	Cephalalgia	8	9	0
Spinal manipulative therapy for chronic low-back pain: an update of a Cochrane review	Rubinstein et al.	2011	Spine	9	26	0
Spinal manipulation for low back pain. An updated systematic review of randomized clinical trials	Koes et al.	1996	Spine	1	46	0
Psychological response in spinal manipulation (PRISM): a systematic review of psychological outcomes in randomised controlled trials	Williams et al.	2007	Complentary Therapy in Medicine	6	28	0
Chiropractic Care for Adults With Pregnancy-Related Low Back, Pelvic Girdle Pain, or Combination Pain: A Systematic Review	Weis et al.	2020	Journal of Manipulative and Physiological Therapeutics	4	3	0
Spinal manipulation for dysmenorrhoea	Proctor et al.	2006	Cochrane Database of Systematic Reviews	7	3	0
Thoracic spine manipulation for the management of mechanical neck pain: A systematic review and meta-analysis	Masaracchio et al.	2019	PLoS One	7	14	0
Spinal manipulations for tension-type headaches: a systematic review of randomized controlled trials	Posadzki et al.	2012	Complementary Therapies in Medicine	8	5	0
Spinal manipulation for the management of cervicogenic headache: A systematic review and meta-analysis	Fernandez et al.	2020	European Journal of Pain	5	6	0
Best Practices for Chiropractic Care for Older Adults: A Systematic Review and Consensus Update	Hawk et al.	2017	Journal of Manipulative and Physiological Therapeutics	6	3	0
Chiropractic spinal manipulation for neck pain: a systematic review	Ernst E	2003	The Journal of Pain	5	4	0
Efficacy of spinal manipulation/mobilization therapy. A meta-analysis	Ottenbacher et al.	1985	Spine	1	9	0
Spinal Manipulation Vs Sham Manipulation for Nonspecific Low Back Pain: A Systematic Review and Meta-analysis	Ruddock et al.	2016	Journal of Chiropractic Medicine	4	9	0
Spinal manipulative therapy for chronic low-back pain	Rubinstein et al.	2011	Cochrane Database of Systematic Reviews	9	45	0
Chiropractic treatment for gastrointestinal problems: a systematic review of clinical trials	Ernst E	2011	Canadian Journal of Gastroenterology	6	2	0
Does cervical spine manipulation reduce pain in people with degenerative cervical radiculopathy? A systematic review of the evidence, and a meta-analysis	Zhu et al.	2016	Clinical Rehabilitation	9	3	1
Evidence-informed management of chronic low back pain with spinal manipulation and mobilization	Bronfort et al.	2008	The Spine Journal	5	27	0
Manipulation or mobilisation for neck pain	Gross et al.	2010	Cochrane Database of Systematic Reviews	6	27	0
Chiropractic treatment for fibromyalgia: a systematic review	Ernst E	2009	Clinical Rheumatology	6	4	0
Manipulative therapy for pregnancy and related conditions: a systematic review	Khorsan et al.	2009	Obstretical and Gynecological Survey	6	1	0
Thoracic spine thrust manipulation improves pain, range of motion, and self-reported function in patients with mechanical neck pain: a systematic review	Cross et al.	2011	Journal of Orthopaedic and Sports Physical Therapy	6	6	0
The Impact of Spinal Manipulation on Migraine Pain and Disability: A Systematic Review and Meta-Analysis	Rist et al.	2019	Headache	2	6	0
Spinal manipulations for the treatment of migraine: a systematic review of randomized clinical trials	Posadzki et al.	2011	Cephalalgia	7	3	0
Spinal manipulation or mobilization for radiculopathy: a systematic review	Leininger et al.	2011	Physical Medicine and Rehabilitation Clinics of North America	5	16	0
Spinal manipulative therapy for low back pain	Assendelft et al.	2004	Cochrane Database of Systematic Reviews	4	52	0
Efficacy of spinal manipulation and mobilization for low back pain and neck pain: a systematic review and best evidence synthesis	Bronfort et al.	2004	The Spine Journal	5	46	1
Does spinal manipulative therapy help people with chronic low back pain?	Ferreira et al.	2002	Australian Journal of Physiotherapy	4	12	0
The effect of spinal manipulative therapy on pain relief and function in patients with chronic low back pain: an individual participant data meta-analysis	de Zoete et al.	2021	Physiotherapy	7	21	0
Manipulation and mobilisation for mechanical neck disorders	Gross et al.	2004	Cochrane Database of Systematic Reviews	6	33	0
NASS Contemporary Concepts in Spine Care: spinal manipulation therapy for acute low back pain	Dagenais et al.	2010	The Spine Journal	1	14	0
Efficacy of spinal manipulative therapy for low back pain of less than three months' duration	Ferreira et al.	2003	Journal of Manipulative and Physiological Therapeutics	4	16	0
Manipulation and Mobilization for Treating Chronic Nonspecific Neck Pain: A Systematic Review and Meta-Analysis for an Appropriateness Panel	Coulter et al.	2019	Pain Physician	6	53	0
Effectiveness of osteopathic interventions in chronic non-specific low back pain: A systematic review and meta-analysis	Dal Farra et al.	2021	Complementary Therapies in Medicine	7	10	0
Manipulative and manual therapies in the management of patients with prior lumbar surgery: A systematic review	Daniels CJ	2021	Complementary Therapies in Clinical Practice	8	7	0

## Appendix 2

See Table 9.

**Table 9:** Overview of all unique randomized controlled trials included

Title	First author	Year of publication	Journal	CINAHL	Cochrane Library	EMBASE	Google Scholar	ICL	MEDLINE/PubMed	Web of Science	PEDro	AMED
Effectiveness of myofascial release in the management of chronic low back pain in nursing professionals	Ajimsha MS	2014	Journal of Bodywork and Movement Therapies	Y	Y	Y	Y	N	Y	N	Y	Y
Role of manual therapy with exercise regime versus exercise regime alone in the management of non-specific chronic neck pain	Akhter S	2014	Pakistan Journal of Pharmaceutical Sciences	N	Y	Y	Y	N	Y	Y	Y	N
Der Einfluss der osteopathischen Behandlung auf Blasenentleerungsstörungen bei Frauen	Alberts K	2005	AFO, Germany	N	N	N	N	N	N	N	N	N
Stretching as an adjunct to chiropractic manipulation of chronic neck pain-before, after or not at all? A prospective randomized controlled clinical trial	Allan M	2003	European Journal of Chiropractic	Y	N	N	N	Y	N	N	Y	Y
A randomized clinical trial of manual therapy for cervico-brachial pain syndrome – a pilot study	Allison GT	2002	Manual Therapy	Y	Y	Y	Y	N	Y	Y	Y	Y
Physical therapy in occipital headaches	Ammer K	1990	Manual Medicine	N	Y	Y	N	N	N	N	Y	Y
A comparison of selected osteopathic treatment and relaxation for tension-type headaches	Anderson RE	2006	Headache	Y	Y	Y	Y	N	Y	Y	Y	N
A comparison of osteopathic spinal manipulation with standard care for patients with low back pain	Andersson GB	1999	New England Journal of Medicine	Y	Y	Y	Y	N	Y	Y	Y	Y
A randomized controlled trial on the effectiveness of a classification-based system for subacute and chronic low back pain	Apeldoorn AT	2012	Spine	Y	Y	Y	Y	N	Y	Y	Y	N
Applying Joint Mobilization at Different Cervical Vertebral Levels does not Influence Immediate Pain Reduction in Patients with Chronic Neck Pain: A Randomized Clinical Trial	Aquino RL	2009	Journal of Manual and Manipulative Therapy	Y	Y	Y	Y	N	Y	N	Y	N
Effects of Myofascial Release in Nonspecific Chronic Low Back Pain: A Randomized Clinical Trial	Arguisuelas MD	2017	Spine	Y	Y	Y	Y	N	Y	Y	Y	N
The efficacy of manual treatment in low back pain: A clinical trial	Arkuszewski Z	1986	Manual Medicine	N	N	N	N	N	N	N	Y	Y
Manual therapy and exercise therapy in patients with chronic low back pain: a randomized, controlled trial with 1-year follow-up	Aure OF	2003	Spine	N	Y	Y	Y	N	Y	Y	Y	Y
Manual therapy followed by specific active exercises versus a placebo followed by specific active exercises on the improvement of functional disability in patients with chronic non specific low back pain: a randomized controlled trial	Balthazard P	2012	BMC Musculoskeletal Disorders	Y	Y	Y	Y	N	Y	Y	Y	N
Manipulative therapy in addition to usual medical care for patients with shoulder dysfunction and pain: a randomized, controlled trial	Bergman GJD	2004	Annals of Internal Medicine	Y	Y	Y	Y	N	Y	Y	Y	N
Acute low back pain in industry. A controlled prospective study with special reference to therapy and confounding factors	Bergquist-Ullman M	1977	Acta Orthopaedica Scandinavica	N	Y	Y	Y	N	Y	Y	Y	N
Efficacy of treating low back pain and dysfunction secondary to osteoarthritis: chiropractic care compared with moist heat alone	Beyerman KL	2006	Journal of Manipulative and Physiological Therapeutics	Y	Y	Y	Y	Y	Y	Y	Y	Y
Spinal manipulative therapy has an immediate effect on thermal pain sensitivity in people with low back pain: a randomized controlled trial	Bialosky JE	2009	Physical Therapy	Y	Y	Y	Y	N	Y	Y	Y	Y
Spinal manipulative therapy-specific changes in pain sensitivity in individuals with low back pain	Bialosky JE	2014	The Journal of Pain	Y	Y	Y	Y	N	Y	Y	Y	N
Immediate effects of a high-velocity spine manipulation in paraspinal muscles activity of nonspecific chronic low-back pain subjects	Bicalho E	2010	Manual Therapy	Y	Y	Y	Y	N	Y	Y	Y	Y
The Chiropractic Hospital-based Interventions Research Outcomes (CHIRO) study: a randomized controlled trial on the effectiveness of clinical practice guidelines in the medical and chiropractic management of patients with acute mechanical low back pain	Bishop PB	2010	The Spine Journal	Y	Y	Y	Y	N	Y	Y	Y	N
Zur Objektivierung der manualtherapeutischen Beeinflußbarkeit des spondylogenen Kopfschmerzes / [Objective criteria for the evaluation of chiropractic treatment of spondylotic headache]	Bitterli J	1977	Der Nervenarzt	N	Y	Y	N	N	Y	Y	Y	N
A controlled, multicentre trial of manual therapy in low-back pain. Initial status, sick-leave and pain score during follow-up	Blomberg S	1992	Scandinavian Journal of Primary Health Care	N	Y	Y	Y	N	Y	N	Y	N
A controlled, multicentre trial of manual therapy with steroid injections in low-back pain: functional variables, side effects and complications during four months follow-up	Blomberg S	1993	Clinical Rehabilitation	Y	Y	Y	Y	N	N	N	Y	Y
Manual therapy with steroid injections in low-back pain. Improvement of quality of life in a controlled trial with four months' follow-up	Blomberg S	1993	Scandinavian Journal of Primary Health Care	N	Y	Y	Y	N	Y	N	Y	Y
A randomized study of manual therapy with steroid injections in low-back pain. Telephone interview follow-up of pain, disability, recovery and drug consumption	Blomberg S	1994	European Spine Journal	N	Y	Y	Y	N	Y	N	Y	N
Manual therapy with steroid injections–a new approach to treatment of low back pain. A controlled multicenter trial with an evaluation by orthopedic surgeons	Blomberg S	1994	Spine	Y	Y	Y	Y	N	Y	Y	Y	N
The effectiveness of chiropractic management of fibromyalgia patients: a pilot study	Blunt KL	1997	Journal of Manipulative and Physiological Therapeutics	Y	Y	Y	Y	Y	Y	Y	Y	Y
Efficacy of high-velocity low-amplitude manipulative technique in subjects with low-back pain during menstrual cramping	Boesler D	1993	Journal of the American Osteopathic Association	N	Y	Y	Y	N	Y	N	Y	Y
Sick leave reductions from a comprehensive manual therapy programme for low back pain: the Gotland Low Back Pain Study	Bogefeldt J	2008	Clinical Rehabilitation	Y	Y	Y	Y	N	Y	Y	N	Y
Spinal manipulation vs. amitriptyline for the treatment of chronic tension-type headaches: a randomized clinical trial	Boline PD	1995	Journal of Manipulative and Physiological Therapeutics	Y	Y	Y	Y	Y	Y	Y	Y	Y
Spinal manipulation in the treatment of episodic tension-type headache: a randomized controlled trial	Bove G	1998	JAMA	Y	Y	Y	Y	N	Y	Y	N	Y
A controlled, prospective pilot study of the possible effects of chiropractic manipulation in the treatment of osteo-arthritis of the hip	Brantingham JW	2003	European Journal of Chiropractic	Y	Y	N	N	Y	N	N	Y	Y
Identifying subgroups of patients with acute/subacute "nonspecific" low back pain: results of a randomized clinical trial	Brennan GP	2006	Spine	Y	Y	Y	Y	N	Y	Y	Y	Y
Immediate effects of inhibitive distraction on active range of cervical flexion in patients with neck pain: a pilot study	Briem K	2007	Journal of Manual and Manipulative Therapy	Y	Y	Y	Y	N	Y	N	Y	N
Cervical pain and mobilization	Brodin H	1984	International Journal of Rehabilitation Research	N	Y	Y	Y	N	Y	Y	Y	N
Chiropractic versus general medical treatment of low back pain: a small scale controlled clinical trial	Bronfort G	1989	American Journal of Chiropractic Medicine	N	Y	N	N	Y	N	N	Y	Y
Trunk exercise combined with spinal manipulative or NSAID therapy for chronic low back pain: a randomized, observer-blinded clinical trial	Bronfort G	1996	Journal of Manipulative and Physiological Therapeutics	Y	Y	Y	Y	Y	Y	N	Y	Y
A randomized clinical trial of exercise and spinal manipulation for patients with chronic neck pain	Bronfort G	2001	Spine	Y	Y	Y	Y	N	Y	Y	Y	Y
Spinal manipulation, epidural injections, and self-care for sciatica: a pilot study for a randomized clinical trial	Bronfort G	2004	Journal of Manipulative and Physiological Therapeutics	Y	Y	Y	Y	Y	Y	Y	Y	Y
Supervised exercise, spinal manipulation, and home exercise for chronic low back pain: a randomized clinical trial	Bronfort G	2011	The Spine Journal	Y	Y	Y	Y	N	Y	Y	Y	Y
Spinal manipulation, medication, or home exercise with advice for acute and subacute neck pain: a randomized trial	Bronfort G	2012	Annals of Internal Medicine	Y	Y	Y	Y	N	Y	Y	Y	Y
Spinal manipulation and home exercise with advice for subacute and chronic back-related leg pain: a trial with adaptive allocation	Bronfort G	2014	Annals of Internal Medicine	Y	Y	Y	Y	N	Y	Y	Y	N
Effectiveness of an extension-oriented treatment approach in a subgroup of subjects with low back pain: a randomized clinical trial	Browder DA	2007	Physical Therapy	Y	Y	Y	Y	N	Y	Y	Y	N
A controlled trial of rotational manipulation in low back pain	Buerger AA	1980	Manual Medicine	N	Y	Y	N	N	N	N	Y	Y
Single-blind randomised controlled trial of chemonucleolysis and manipulation in the treatment of symptomatic lumbar disc herniation	Burton AK	2000	European Spine Journal	N	Y	Y	Y	N	Y	Y	Y	Y
Randomized controlled trial of specific spinal stabilization exercises and conventional physiotherapy for recurrent low back pain	Cairns MC	2006	Spine	Y	Y	Y	Y	N	Y	Y	Y	Y
Amount of health care and self-care following a randomized clinical trial comparing flexion-distraction with exercise program for chronic low back pain	Cambron JA	2006	Chiropractic and Osteopathy	Y	Y	Y	Y	Y	Y	N	Y	N
One-year follow-up of a randomized clinical trial comparing flexion distraction with an exercise program for chronic low-back pain	Cambron JA	2006	Journal of Alternative and Complementary Medicine	Y	Y	Y	Y	N	Y	Y	Y	Y
Muscle tenderness in tension headache treated with acupuncture or physiotherapy	Carlsson J	1990	Cephalalgia	N	Y	Y	Y	N	Y	Y	Y	N
The access randomized clinical trial of public versus private physiotherapy for low back pain	Casserley-Feeney SN	2012	Spine	Y	Y	Y	Y	N	Y	Y	Y	N
The immediate effect of manipulation versus mobilization on pain and range of motion in the cervical spine: a randomized controlled trial	Cassidy JD	1992	Journal of Manipulative and Physiological Therapeutics	N	Y	Y	Y	Y	Y	Y	Y	Y
Effectiveness of manual therapy compared to usual care by the general practitioner for chronic tension-type headache: design of a randomised clinical trial	Castien RF	2009	BMC Musculoskeletal Disorders	Y	Y	Y	Y	N	Y	Y	Y	Y
Benefits of Craniosacral Therapy in Patients with Chronic Low Back Pain: A Randomized Controlled Trial	Castro-Sánchez AM	2016	Journal of Complementary Medicine	Y	Y	Y	Y	N	Y	Y	Y	N
Short-term effectiveness of spinal manipulative therapy versus functional technique in patients with chronic nonspecific low back pain: a pragmatic randomized controlled trial	Castro-Sánchez AM	2016	The Spine Journal	Y	Y	Y	Y	N	Y	Y	Y	N
Spinal manipulation compared with back school and with individually delivered physiotherapy for the treatment of chronic low back pain: a randomized trial with one-year follow-up	Cecchi F	2010	Clinical Rehabilitation	Y	Y	Y	Y	N	Y	Y	Y	Y
Predictors of functional outcome in patients with chronic low back pain undergoing back school, individual physiotherapy or spinal manipulation	Cecchi F	2012	European Journal of Physical and Rehabilitation Medicine	Y	Y	Y	Y	N	Y	Y	Y	Y
Clinical effectiveness of osteopathic treatment in chronic migraine: 3-Armed randomized controlled trial	Cerritelli F	2015	Complementary Therapies in Medicine	Y	Y	Y	Y	N	Y	Y	N	N
Chiropractic spinal manipulative therapy for cervicogenic headache: a single-blinded, placebo, randomized controlled trial	Chaibi A	2017	BMC Research Notes	N	Y	Y	Y	N	Y	N	Y	N
Chiropractic spinal manipulative therapy for migraine: a three-armed, single-blinded, placebo, randomized controlled trial	Chaibi A	2017	European Journal of Neurology	N	Y	Y	Y	N	Y	Y	N	N
[Clinical research on the fine tuning manipulation of short lever on spine in treating patients with cervical spondylotic myelopathy]	Chen J	2009	Chin J Tradit Med Trauma Orthop	N	N	N	N	N	N	N	Y	Y
A comparison of physical therapy, chiropractic manipulation, and provision of an educational booklet for the treatment of patients with low back pain	Cherkin DC	1998	New England Journal of Medicine	Y	Y	Y	Y	N	Y	Y	Y	Y
A clinical prediction rule to identify patients with low back pain most likely to benefit from spinal manipulation: a validation study	Childs JD	2004	Annals of Internal Medicine	Y	Y	Y	Y	N	Y	Y	N	Y
A perspective for considering the risks and benefits of spinal manipulation in patients with low back pain	Childs JD	2006	Manual Therapy	Y	Y	Y	Y	N	Y	Y	N	N
A prospective study of patients with chronic back pain randomised to group exercise, physiotherapy or osteopathy	Chown M	2008	Physiotherapy	Y	Y	Y	Y	N	N	Y	Y	N
Menopausal symptoms: an osteopathic investigation	Cleary C	1994	Complementary Therapies in Medicine	Y	Y	Y	Y	N	N	N	Y	N
Short-Term Effects of Thoracic Manipulation on Lower Trapezius Muscle Strength	Cleland JA	2004	Journal of Manual and Manipulative Therapy	Y	Y	Y	Y	N	N	N	Y	Y
Immediate effects of thoracic manipulation in patients with neck pain: a randomized clinical trial	Cleland JA	2005	Manual Therapy	Y	Y	Y	Y	N	Y	Y	Y	Y
Short-term effects of thrust versus nonthrust mobilization/manipulation directed at the thoracic spine in patients with neck pain: a randomized clinical trial	Cleland JA	2007	Physical Therapy	Y	Y	Y	Y	N	Y	Y	Y	N
Comparison of the effectiveness of three manual physical therapy techniques in a subgroup of patients with low back pain who satisfy a clinical prediction rule: a randomized clinical trial	Cleland JA	2009	Spine	Y	Y	Y	Y	N	Y	Y	Y	Y
Examination of a clinical prediction rule to identify patients with neck pain likely to benefit from thoracic spine thrust manipulation and a general cervical range of motion exercise: multi-center randomized clinical trial	Cleland JA	2010	Physical Therapy	Y	Y	Y	Y	N	Y	Y	Y	Y
Early use of thrust manipulation versus non-thrust manipulation: a randomized clinical trial	Cook C	2013	Manual Therapy	Y	Y	Y	Y	N	Y	Y	N	N
Can a within/between-session change in pain during reassessment predict outcome using a manual therapy intervention in patients with mechanical low back pain?	Cook CE	2012	Manual Therapy	Y	Y	Y	Y	N	Y	Y	Y	Y
Which prognostic factors for low back pain are generic predictors of outcome across a range of recovery domains?	Cook CE	2013	Physical Therapy	Y	Y	Y	Y	N	Y	Y	Y	Y
The immediate effects of manual therapy in patients with cervicobrachial pain on neural origin: a pilot study	Coppieters MW	2000	IFOMT 2000: International Federation of Orthopaedic Manipulative Therapists in Conjunction With the 11th Biennial Conference of the Manipulative Physiotherapists Association of Australia. Perth: The University of Western Australia	N	Y	N	N	N	N	N	Y	Y
Aberrant protective force generation during neural provocation testing and the effect of treatment in patients with neurogenic cervicobrachial pain	Coppieters MW	2003	Journal of Manipulative and Physiological Therapeutics	Y	Y	Y	Y	Y	Y	Y	Y	Y
The immediate effects of a cervical lateral glide treatment technique in patients with neurogenic cervicobrachial pain	Coppieters MW	2003	Journal of Orthopaedic and Sports Physical Therapy	Y	Y	Y	Y	N	Y	Y	Y	Y
Multicentre trial of physiotherapy in the management of sciatic symptoms	Coxhead CE	1981	Lancet	N	Y	Y	Y	N	Y	Y	N	Y
Low back pain treated by manipulation; a controlled series	Coyer AB	1955	British Medical Journal	N	Y	Y	Y	N	Y	Y	N	Y
The Hmax/Mmax ratio as an outcome measure for acute low back pain	Cramer GD	1993	Journal of Manipulative and Physiological Therapeutics	N	Y	Y	Y	Y	Y	Y	N	N
A randomized, controlled trial of osteopathic manipulative treatment for acute low back pain in active duty military personnel	Cruser A	2012	Journal of Manual and Manipulative Therapy	Y	Y	Y	Y	N	Y	N	Y	Y
Osteopathic manual therapy versus conventional conservative therapy in the treatment of temporomandibular disorders: a randomized controlled trial	Cuccia AM	2010	Journal of Bodywork and Movement Therapies	Y	Y	Y	Y	N	Y	N	Y	Y
Exercise, manual therapy, and education with or without high-intensity deep-water running for nonspecific chronic low back pain: a pragmatic randomized controlled trial	Cuesta-Vargas AI	2011	American Journal of Physical Medicine and Rehabilitation	N	Y	Y	Y	N	Y	Y	Y	N
Training primary care physicians to give limited manual therapy for low back pain: patient outcomes	Curtis P	2000	Spine	N	Y	Y	Y	N	Y	Y	Y	N
Chronic neck pain: a comparison of acupuncture treatment and physiotherapy	David J	1998	British Journal of Rheumatology	N	Y	Y	Y	N	Y	Y	Y	Y
Immediate effects of region-specific and non-region-specific spinal manipulative therapy in patients with chronic low back pain: a randomized controlled trial	de Oliveira RF	2013	Physical Therapy	Y	Y	Y	Y	N	Y	Y	Y	N
Evidence for use of an extension-mobilization category in acute low back syndrome: a prescriptive validation pilot study	Delitto A	1993	Physical Therapy	Y	Y	Y	Y	N	Y	Y	Y	Y
Results of two different manual therapy techniques in chronic tension-type headache	Demirturk F	2002	The Pain Clinic	N	Y	Y	Y	N	N	Y	Y	N
Efficacy of preventive spinal manipulation for chronic low-back pain and related disabilities: a preliminary study	Descarreaux M	2004	Journal of Manipulative and Physiological Therapeutics	Y	Y	Y	Y	Y	Y	Y	Y	Y
A Study to Determine the Effectiveness of Spinal Manipulative Therapy Versus Manipulative Therapy of the Glenohumeral Joint Treatment of Impingement Syndrome [Master Thesis]	Deverneuil V	2002	University of Johannesburg	N	N	N	Y	N	N	N	Y	Y
Clinical effectiveness analysis of Tuina manipulation for treating lumbar disc herniation	Ding Y	2014	Chin J Clin Ration Drug Use	N	N	N	N	N	N	N	Y	Y
The role of patients' expectation of appropriate initial manual therapy treatment in outcomes for patients with low back pain	Donaldson M	2013	Journal of Manipulative and Physiological Therapeutics	Y	Y	Y	Y	Y	Y	Y	Y	Y
Manipulation in treatment of low back pain: a multicentre study	Doran DM	1975	British Medical Journal	N	Y	Y	Y	N	Y	Y	Y	Y
Evaluation of a modified clinical prediction rule for use with spinal manipulative therapy in patients with chronic low back pain: a randomized clinical trial	Dougherty PE	2014	Chiropractic and Manual Therapies	N	Y	Y	Y	Y	Y	N	Y	Y
Spinal Manipulative Therapy for Chronic Lower Back Pain in Older Veterans: A Prospective, Randomized, Placebo-Controlled Trial	Dougherty PE	2014	Geriatric Orthopaedic Surgery and Rehabilitation	N	Y	Y	Y	N	Y	Y	N	N
Effectiveness of osteopathy in the cranial field and myofascial release versus acupuncture as complementary treatment for children with spastic cerebral palsy: a pilot study	Duncan B	2008	Journal of the American Osteopathic Association	Y	Y	Y	Y	N	Y	N	N	N
Upper cervical and upper thoracic thrust manipulation versus nonthrust mobilization in patients with mechanical neck pain: a multicenter randomized clinical trial	Dunning JR	2012	Journal of Orthopaedic and Sports Physical Therapy	Y	Y	Y	Y	N	Y	Y	Y	Y
Upper cervical and upper thoracic manipulation versus mobilization and exercise in patients with cervicogenic headache: a multi-center randomized clinical trial	Dunning JR	2016	BMC Musculoskeletal Disorders	Y	Y	Y	Y	N	Y	Y	Y	N
Effectiveness of manual therapy or pulsed shortwave diathermy in addition to advice and exercise for neck disorders: a pragmatic randomized controlled trial in physical therapy clinics	Dziedzic K	2005	Arthritis and Rheumatism	Y	Y	Y	Y	N	Y	Y	N	N
Relative Therapeutic Efficacy of Some Vertebral Mobilization Techniques in the Management of Unilateral Cervical Spondylosis: A Comparative Study	Egwu MO	2008	Journal of Physical Therapy Science	N	Y	N	Y	N	N	Y	Y	N
Effect of Adding Neural Mobilization Versus Myofascial Release to Stabilization Exercises after Lumbar Spine Fusion: A Randomized Controlled Trial	Elsayyad MM	2021	Archives of Physical Medicine and Rehabilitation	Y	Y	Y	Y	N	Y	Y	Y	Y
Satisfaction of patients with mechanical neck disorders attended to by primary care physical therapists	Elustondo SG	2010	Journal of Evaluation in Clinical Practice	Y	Y	Y	Y	N	Y	Y	Y	Y
A randomized controlled trial of chiropractic compared to physical therapy for chronic low back pain in community dwelling geriatric patients	Enix DE	2015	Top Integr Health Care	N	N	N	Y	Y	N	N	Y	N
Relative effectiveness of an extension program and a combined program of manipulation and flexion and extension exercises in patients with acute low back syndrome	Erhard RE	1994	Physical Therapy	Y	Y	Y	Y	N	Y	Y	Y	Y
Studie zur Behandlung der Dranginkontinenz und der Kombination aus Stress- und Dranginkontinenz bei Frauen	Ernst H	2002	AFO, Germany	N	N	N	N	N	N	N	N	Y
[Randomized clinical trial for primary care patients with neck pain: manual therapy versus electrical stimulation]	Escortell Mayor E	2008	Atencion Primaria	N	Y	Y	Y	N	Y	Y	Y	Y
Primary care randomized clinical trial: manual therapy effectiveness in comparison with TENS in patients with neck pain	Escortell Mayor E	2011	Manual Therapy	Y	Y	Y	Y	N	Y	Y	N	N
Lumbar spinal manipulation on trial. Part I–clinical assessment	Evans DP	1978	Rheumatology and Rehabilitation	N	Y	Y	Y	N	Y	Y	N	N
Two-year follow-up of a randomized clinical trial of spinal manipulation and two types of exercise for patients with chronic neck pain	Evans R	2002	Spine	Y	Y	Y	Y	N	Y	Y	Y	Y
Supervised exercise with and without spinal manipulation performs similarly and better than home exercise for chronic neck pain: a randomized controlled trial	Evans R	2012	Spine	Y	Y	Y	Y	N	Y	Y	N	N
Acute low back pain. Comparison of two conservative treatment approaches	Farrell JP	1982	Medical Journal of Australia	N	Y	Y	Y	N	Y	Y	Y	N
Differential recruitment in a cluster randomized trial in primary care: the experience of the UK back pain, exercise, active management and manipulation (UK BEAM) feasibility study	Farrin A	2005	Clinical Trials	N	Y	Y	Y	N	Y	Y	Y	Y
Immediate Hypoalgesic and Motor Effects After a Single Cervical Spine Manipulation in Subjects With Lateral Epicondylalgia	Fernández-Carnero J	2008	Journal of Manipulative and Physiological Therapeutics	Y	Y	Y	Y	Y	Y	Y	Y	N
Examination of Motor and Hypoalgesic Effects of Cervical vs Thoracic Spine Manipulation in Patients With Lateral Epicondylalgia: A Clinical Trial	Fernández-Carnero J	2011	Journal of Manipulative and Physiological Therapeutics	Y	Y	Y	Y	Y	Y	Y	Y	N
Dorsal Manipulation in Whiplash Injury Treatment: A Randomized Controlled Trial	Fernández-de-Las-Peñas C	2004	Journal of Whiplash and Related Disorders	Y	Y	Y	Y	N	N	N	Y	N
Changes in pressure pain thresholds over C5-C6 zygapophyseal joint after a cervicothoracic junction manipulation in healthy subjects	Fernández-de-Las-Peñas C	2008	Journal of Manipulative and Physiological Therapeutics	Y	Y	Y	Y	Y	Y	Y	Y	N
Repeated Applications of Thoracic Spine Thrust Manipulation do not Lead to Tolerance in Patients Presenting with Acute Mechanical Neck Pain: A Secondary Analysis	Fernández-de-Las-Peñas C	2009	Journal of Manual and Manipulative Therapy	Y	Y	Y	Y	N	Y	N	N	N
Does spinal manipulative therapy help people with chronic low back pain?	Ferreira ML	2002	Australian Journal of Physiotherapy	Y	Y	Y	Y	N	Y	Y	Y	N
Comparison of general exercise, motor control exercise and spinal manipulative therapy for chronic low back pain: A randomized trial	Ferreira ML	2007	Pain	Y	Y	Y	Y	N	Y	Y	Y	Y
Changes in recruitment of transversus abdominis correlate with disability in people with chronic low back pain	Ferreira PH	2010	British Journal of Sports Medicine	Y	Y	Y	Y	N	Y	Y	Y	N
Efficacy of classification-based cognitive functional therapy in patients with non-specific chronic low back pain: a randomized controlled trial	Fersum KV	2013	European Journal of Pain	Y	Y	Y	Y	N	Y	Y	N	Y
A controlled trial of manipulation in a selected group of patients with low back pain favouring one side	Fisk JW	1979	New Zealand Medical Journal	N	N	Y	Y	Y	Y	Y	Y	N
Osteopathy improves the severity of irritable bowel syndrome: a pilot randomized sham-controlled study	Florance BM	2012	European Journal of Gastroenterology and Hepatology	Y	Y	Y	Y	N	Y	Y	Y	N
Early Physical Therapy vs Usual Care in Patients With Recent-Onset Low Back Pain: A Randomized Clinical Trial	Fritz JM	2015	JAMA	Y	Y	Y	Y	N	Y	Y	Y	Y
Randomised controlled trial of physiotherapy compared with advice for low back pain	Frost H	2004	BMJ	Y	Y	Y	Y	N	Y	Y	Y	N
Osteopathic manipulative treatment in conjunction with medication relieves pain associated with fibromyalgia syndrome: results of a randomized clinical pilot project	Gamber RG	2002	Journal of the American Osteopathic Association	Y	Y	Y	Y	N	Y	N	Y	Y
A randomized, controlled trial of manual therapy and specific adjuvant exercise for chronic low back pain	Geisser ME	2005	The Clinical Journal of Pain	N	Y	Y	Y	N	Y	Y	Y	N
[Osteopathic versus orthopedic treatments for chronic epicondylopathia humeri radialis: a randomized controlled trial]	Geldschläger S	2004	Forschende Komplementärmedizin und klassische Naturheilkunde	N	Y	Y	Y	N	Y	Y	N	N
Relative immediate effect of ischaemic compression and activator trigger point therapy on active upper trapezius trigger points: a randomised trial	Gemmell H	2008	Clinical Chiropractic	Y	Y	N	Y	Y	N	N	N	N
Relative effectiveness and adverse effects of cervical manipulation, mobilisation and the activator instrument in patients with sub-acute non-specific neck pain: results from a stopped randomised trial	Gemmell H	2010	Chiropractic and Osteopathy	Y	Y	Y	Y	Y	Y	N	Y	Y
The immediate effect of activator vs. meric adjustment on acute low back pain: a randomized controlled trial	Gemmell HA	1995	Journal of Manipulative and Physiological Therapeutics	Y	Y	Y	Y	Y	Y	Y	Y	N
Osteopathische Behandlung von Frauen mit Harninkontinenz mit Verletzung des Perineumscunter der Entbindung	Gerhardt K	2005	AFO, Germany	N	N	N	N	N	N	N	Y	Y
[Chronic low back pain and vertebral manipulation]	Ghroubi S	2007	Annals de Réadaptation et de Médecine Physique	N	Y	Y	Y	N	Y	N	Y	Y
Controlled comparison of short-wave diathermy treatment with osteopathic treatment in non-specific low back pain	Gibson T	1985	Lancet	N	Y	Y	Y	N	Y	Y	N	Y
**Neck sprain: Physiotherapy vs collar treatment. Die Distorsion der Halswirbelsäule: frühfunktionelle vs. ruhigstellende Behandlung**	Giebel GD	1997	Zentralblatt für Chirurgie	N	Y	Y	Y	N	Y	N	Y	Y
Chronic spinal pain syndromes: a clinical pilot trial comparing acupuncture, a nonsteroidal anti-inflammatory drug, and spinal manipulation	Giles LG	1999	Journal of Manipulative and Physiological Therapeutics	Y	Y	Y	Y	Y	Y	Y	Y	N
Chronic spinal pain: a randomized clinical trial comparing medication, acupuncture, and spinal manipulation	Giles LG	2003	Spine	Y	Y	Y	Y	N	Y	Y	Y	Y
A clinical trial of rotational manipulation of the spine in back pain cases occurring in a factory	Glover JR	1966	Proceedings of the Royal Society of Medicine	N	Y	Y	Y	N	Y	Y	N	N
Occupational health research and the problem of back pain	Glover JR	1971	Transactions of the Society of Occupational Medicine	N	Y	Y	Y	N	Y	N	Y	N
Back pain: a randomized clinical trial of rotational manipulation of the trunk	Glover JR	1974	British Journal of Industrial Medicine	N	Y	Y	Y	N	Y	Y	Y	N
A randomized trial of manipulation for low-back pain in a medical setting	Godfrey CM	1984	Spine	Y	Y	Y	Y	N	Y	Y	Y	Y
Adding chiropractic manipulative therapy to standard medical care for patients with acute low back pain: results of a pragmatic randomized comparative effectiveness study	Goertz CM	2013	Spine	Y	Y	Y	Y	N	Y	Y	Y	N
A randomized controlled trial investigating the efficiency of musculoskeletal physiotherapy on chronic low back disorder	Goldby LJ	2006	Spine	Y	Y	Y	Y	N	Y	Y	Y	N
The impact of treatment confidence on pain and related disability among patients with low-back pain: results from the University of California, Los Angeles, low-back pain study	Goldstein MS	2002	The Spine Journal	N	Y	Y	Y	N	Y	N	Y	Y
Thoracic spine manipulation for the management of patients with neck pain: a randomized clinical trial	González-Iglesias J	2009	Journal of Orthopaedic and Sports Physical Therapy	Y	Y	Y	Y	N	Y	Y	Y	Y
Inclusion of thoracic spine thrust manipulation into an electro-therapy/thermal program for the management of patients with acute mechanical neck pain: a randomized clinical trial	González-Iglesias J	2009	Manual Therapy	Y	Y	Y	Y	N	Y	Y	N	N
Short-term effects of lumbar posteroanterior mobilization in individuals with low-back pain	Goodsell M	2000	Journal of Manipulative and Physiological Therapeutics	Y	Y	Y	Y	Y	Y	Y	N	N
A randomized controlled clinical trial of stay-active care versus manual therapy in addition to stay-active care: functional variables and pain	Grunnesjö MI	2004	Journal of Manipulative and Physiological Therapeutics	Y	Y	Y	Y	Y	Y	Y	Y	N
A randomized controlled trial of the effects of muscle stretching, manual therapy and steroid injections in addition to 'stay active' care on health-related quality of life in acute or subacute low back pain	Grunnesjö MI	2011	Clinical Rehabilitation	Y	Y	Y	Y	N	Y	Y	Y	N
A Randomized Clinical Trial Comparing Flexion-Distraction With Active Exercise For Chronic Low Back Pain: A Feasibility Study	Gudavalli MR	2004	Journal of Chiropractic Education	N	Y	N	N	Y	N	N	Y	N
A randomized clinical trial and subgroup analysis to compare flexion-distraction with active exercise for chronic low back pain	Gudavalli MR	2006	European Spine Journal	N	Y	Y	Y	N	Y	Y	Y	Y
Self-management of persistent neck pain: a randomized controlled trial of a multi-component group intervention in primary health care	Gustavsson C	2010	European Journal of Pain	Y	Y	Y	Y	N	Y	Y	Y	N
Effect of pressure applied to the upper thoracic (placebo) versus lumbar areas (osteopathic manipulative treatment) for inhibition of lumbar myalgia during labor	Guthrie RA	1982	Journal of the American Osteopathic Association	N	Y	Y	Y	N	Y	Y	N	Y
Efficacy of cervical endplay assessment as an indicator for spinal manipulation	Haas M	2003	Spine	Y	Y	Y	Y	N	Y	Y	Y	Y
Dose response for chiropractic care of chronic cervicogenic headache and associated neck pain: a randomized pilot study	Haas M	2004	Journal of Manipulative and Physiological Therapeutics	Y	Y	Y	Y	Y	Y	Y	Y	Y
Dose–response for chiropractic care of chronic low back pain	Haas M	2004	The Spine Journal	N	Y	Y	Y	N	Y	Y	Y	N
Dose response and efficacy of spinal manipulation for chronic cervicogenic headache: a pilot randomized controlled trial	Haas M	2010	The Spine Journal	Y	Y	Y	Y	N	Y	Y	Y	Y
Dose–response and efficacy of spinal manipulation for care of chronic low back pain: a randomized controlled trial	Haas M	2014	The Spine Journal	Y	Y	Y	Y	N	Y	Y	N	N
Dose–response and efficacy of spinal manipulation for care of cervicogenic headache: a dual-center randomized controlled trial	Haas M	2018	The Spine Journal	Y	Y	Y	Y	N	Y	Y	Y	Y
A benefit of spinal manipulation as adjunctive therapy for acute low-back pain: a stratified controlled trial	Hadler NM	1987	Spine	N	Y	Y	Y	N	Y	Y	Y	N
Scapular kinematics pre- and post-thoracic thrust manipulation in individuals with and without shoulder impingement symptoms: a randomized controlled study	Haik MN	2014	Journal of Orthopaedic and Sports Physical Therapy	Y	Y	Y	Y	N	Y	Y	N	N
Gastroesophageal Reflux Disease, Spinal Manipulative Therapy and Ischemic Compression: A Preliminary Study	Hains G	2007	Journal of the American Chiropractic Association	Y	Y	N	Y	Y	N	N	Y	Y
Efficacy of a C1-C2 self-sustained natural apophyseal glide (SNAG) in the management of cervicogenic headache	Hall T	2007	Journal of Orthopaedic and Sports Physical Therapy	Y	Y	Y	Y	N	Y	Y	Y	N
Manipulative therapy and clinical prediction criteria in treatment of acute nonspecific low back pain	Hallegraeff JM	2009	Perceptual and Motor Skills	Y	Y	Y	Y	N	Y	Y	Y	N
Manipulative therapy and/or NSAIDs for acute low back pain: design of a randomized controlled trial [ACTRN012605000036617] (study protocol)	Hancock MJ	2005	BMC Musculoskeletal Disorders	N	Y	Y	Y	N	Y	Y	Y	N
Assessment of diclofenac or spinal manipulative therapy, or both, in addition to recommended first-line treatment for acute low back pain: a randomised controlled trial	Hancock MJ	2007	Lancet	Y	Y	Y	Y	N	Y	Y	Y	N
The effectiveness of spinal manipulative therapy versus manipulation of the acromioclavicular joint in the treatment of impingement syndrome of the shoulder [Master Thesis]	Hari M	2008	University of Johannesburg	N	N	N	N	N	N	N	N	N
Preliminary study of the effects of a placebo chiropractic treatment with sham adjustments	Hawk C	1999	Journal of Manipulative and Physiological Therapeutics	Y	Y	Y	Y	Y	Y	Y	Y	Y
Issues in planning a placebo-controlled trial of manual methods: results of a pilot study	Hawk C	2002	Journal of Alternative and Complementary Medicine	Y	Y	Y	Y	N	Y	Y	Y	Y
A randomized trial investigating a chiropractic manual placebo: a novel design using standardized forces in the delivery of active and control treatments	Hawk C	2005	Journal of Alternative and Complementary Medicine	Y	Y	Y	Y	N	Y	Y	Y	Y
Study on the Analgesic Effect of Acupuncture Plus Long's Manipulation on Nerve root-type Cervical Spondylopathy	He JT	2007	Shanghai Journal of Acupuncture and Moxibustion	N	N	N	N	N	N	N	N	N
Manipulation in low back pain	Helliwell PS	1987	The Physician	N	Y	N	N	N	N	N	Y	Y
Does folk medicine work? A randomized clinical trial on patients with prolonged back pain	Hemmilä HM	1997	Archives of Physical Medicine and Rehabilitation	Y	Y	Y	Y	N	Y	Y	Y	Y
Long-term effectiveness of bone-setting, light exercise therapy, and physiotherapy for prolonged back pain: a randomized controlled trial	Hemmilä HM	2002	Journal of Manipulative and Physiological Therapeutics	Y	Y	Y	Y	Y	Y	Y	N	N
Bone setting for prolonged neck pain: a randomized clinical trial	Hemmilä HM	2005	Journal of Manipulative and Physiological Therapeutics	Y	Y	Y	Y	Y	Y	Y	Y	N
Pregnancy Research on Osteopathic Manipulation Optimizing Treatment Effects: the PROMOTE study	Hensel KL	2015	American Journal of Obstetrics and Gynecology	Y	Y	Y	Y	N	Y	Y	N	N
Comparing the satisfaction of low back pain patients randomized to receive medical or chiropractic care: results from the UCLA low-back pain study	Hertzman-Miller RP	2002	American Journal of Public Health	Y	Y	Y	Y	N	Y	Y	Y	Y
Effects of different treatment modalities on gait symmetry and clinical measures for sacroiliac joint patients	Herzog W	1991	Journal of Manipulative and Physiological Therapeutics	N	Y	Y	Y	Y	Y	Y	Y	Y
Spinal high-velocity low amplitude manipulation in acute nonspecific low back pain: a double-blinded randomized controlled trial in comparison with diclofenac and placebo	Heymann WJ	2013	Spine	Y	Y	Y	Y	N	Y	Y	Y	Y
Short-term effects of Mulligan mobilization with movement on pain, disability, and kinematic spinal movements in patients with nonspecific low back pain: a randomized placebo-controlled trial	Hidalgo B	2015	Journal of Manipulative and Physiological Therapeutics	Y	Y	Y	Y	Y	Y	Y	N	N
Spinal manipulation for low back pain	Hoehler FK	1981	JAMA	N	Y	Y	Y	N	Y	Y	Y	N
Westeinde sciatica trial: randomized controlled study of bed rest and physiotherapy for acute sciatica	Hofstee DJ	2002	Journal of Neurosurgery	N	Y	Y	Y	N	Y	Y	Y	Y
A randomized clinical trial comparing chiropractic to conservative medical care for subacute low back pain	Hoiriis KT	2002	European Journal of Chiropractic	Y	Y	N	N	Y	N	N	Y	Y
A randomized clinical trial comparing chiropractic adjustments to muscle relaxants for subacute low back pain	Hoiriis KT	2004	Journal of Manipulative and Physiological Therapeutics	Y	Y	Y	Y	Y	Y	Y	Y	Y
Spinal manipulative therapy versus a low force mimic maneuver for women with primary dysmenorrhea: a randomized, observer-blinded, clinical trial	Hondras MA	1999	Pain	Y	Y	Y	Y	N	Y	Y	Y	N
Recruitment and enrollment for the simultaneous conduct of 2 randomized controlled trials for patients with subacute and chronic low back pain at a CAM research center	Hondras MA	2008	Journal of Alternative and Complementary Medicine	Y	Y	Y	Y	N	Y	Y	Y	N
A randomized controlled trial comparing 2 types of spinal manipulation and minimal conservative medical care for adults 55 years and older with subacute or chronic low back pain	Hondras MA	2009	Journal of Manipulative and Physiological Therapeutics	Y	Y	Y	Y	Y	Y	Y	Y	Y
A comparison of manual therapy and active rehabilitation in the treatment of non specific low back pain with particular reference to a patient's Linton and Hallden psychological screening score: a pilot study	Hough E	2007	BMC Musculoskeletal Disorders	N	Y	Y	Y	Y	Y	Y	Y	N
Neck Pain in Primary Care. The effects of commonly applied interventions	Hoving JL	2001	Institute for Research in Extramural Medicine (EMGO Institute) of the Vrije Universiteit, The Netherlands	N	N	N	N	N	N	N	Y	Y
Manual therapy, physical therapy, or continued care by a general practitioner for patients with neck pain. A randomized, controlled trial	Hoving JL	2002	Annals of Internal Medicine	Y	Y	Y	Y	N	Y	Y	Y	N
Manual therapy, physical therapy, or continued care by the general practitioner for patients with neck pain: long-term results from a pragmatic randomized clinical trial	Hoving JL	2006	The Clinical Journal of Pain	Y	Y	Y	Y	N	Y	Y	Y	N
Manipulation of the cervical spine–a pilot study	Howe DH	1983	Journal of the Royal College of General Practitioners	N	Y	Y	Y	N	Y	Y	N	Y
Osteopathic manipulation in the treatment of muscle-contraction headache	Hoyt WH	1979	Journal of the American Osteopathic Association	N	Y	Y	N	N	Y	Y	Y	N
Functional outcomes of low back pain: comparison of four treatment groups in a randomized controlled trial	Hsieh CY	1992	Journal of Manipulative and Physiological Therapeutics	N	Y	Y	Y	Y	Y	Y	Y	N
Effectiveness of four conservative treatments for subacute low back pain: a randomized clinical trial	Hsieh CY	2002	Spine	Y	Y	Y	Y	N	Y	Y	N	N
Treatment of low back pain by acupressure and physical therapy: randomised controlled trial	Hsieh LL	2006	BMJ	Y	Y	Y	Y	N	Y	Y	Y	Y
Treatment of irritable bowel syndrome with osteopathy: results of a randomized controlled pilot study	Hundscheid HW	2007	Journal of Gastroenterology and Hepatology	N	Y	Y	Y	N	Y	Y	Y	N
A randomized clinical trial of manipulative therapy and interferential therapy for acute low back pain	Hurley DA	2004	Spine	Y	Y	Y	Y	N	Y	Y	N	N
A descriptive study of the usage of spinal manipulative therapy techniques within a randomized clinical trial in acute low back pain	Hurley DA	2005	Manual Therapy	Y	Y	Y	Y	N	Y	Y	Y	Y
A randomized trial of chiropractic manipulation and mobilization for patients with neck pain: clinical outcomes from the UCLA neck-pain study	Hurwitz EL	2002	American Journal of Public Health	Y	Y	Y	Y	N	Y	Y	Y	N
(Second Prize:) The effectiveness of physical modalities among patients with low back pain randomized to chiropractic care: findings from the UCLA low back pain study	Hurwitz EL	2002	Journal of Manipulative and Physiological Therapeutics	Y	Y	Y	Y	Y	Y	Y	Y	N
A randomized trial of medical care with and without physical therapy and chiropractic care with and without physical modalities for patients with low back pain: 6-month follow-up outcomes from the UCLA low back pain study	Hurwitz EL	2002	Spine	Y	Y	Y	Y	N	Y	Y	Y	N
Cross-sectional and longitudinal associations of low-back pain and related disability with psychological distress among patients enrolled in the UCLA Low-Back Pain Study	Hurwitz EL	2003	Journal of Clinical Epidemiology	N	Y	Y	Y	N	Y	Y	Y	Y
Adverse reactions to chiropractic treatment and their effects on satisfaction and clinical outcomes among patients enrolled in the UCLA Neck Pain Study	Hurwitz EL	2004	Journal of Manipulative and Physiological Therapeutics	Y	Y	Y	Y	Y	Y	Y	Y	Y
Effects of recreational physical activity and back exercises on low back pain and psychological distress: findings from the UCLA Low Back Pain Study	Hurwitz EL	2005	American Journal of Public Health	Y	Y	Y	Y	N	Y	Y	Y	Y
Satisfaction as a predictor of clinical outcomes among chiropractic and medical patients enrolled in the UCLA low back pain study	Hurwitz EL	2005	Spine	Y	Y	Y	Y	N	Y	Y	Y	Y
Frequency and clinical predictors of adverse reactions to chiropractic care in the UCLA neck pain study	Hurwitz EL	2005	Spine	Y	Y	Y	Y	N	Y	Y	N	N
The impact of psychosocial factors on neck pain and disability outcomes among primary care patients: results from the UCLA Neck Pain Study	Hurwitz EL	2006	Disability and Rehabilitation	Y	Y	Y	Y	N	Y	Y	Y	Y
A randomized trial of chiropractic and medical care for patients with low back pain: eighteen-month follow-up outcomes from the UCLA low back pain study	Hurwitz EL	2006	Spine	Y	Y	Y	Y	N	Y	Y	N	Y
Is one better than another?: A randomized clinical trial of manual therapy for patients with chronic neck pain	Izquierdo Péres H	2014	Manual Therapy	Y	Y	Y	Y	N	Y	Y	Y	N
Mobilization and manipulation for low-back pain	Jayson MI	1981	Spine	N	Y	Y	Y	N	Y	Y	Y	Y
The value of intermittent cervical traction in recent cervical radiculopathy	Jellad A	2009	Annals of Physical and Rehabilitation Medicine	N	Y	Y	Y	N	Y	N	Y	N
An open study comparing manual therapy with the use of cold packs in the treatment of post-traumatic headache	Jensen OK	1990	Cephalalgia	N	Y	Y	Y	N	Y	Y	Y	Y
[Efficacy of cervical fixed-point traction manipulation for cervical spondylotic radiculopathy: a randomized controlled trial]	Jiang CB	2012	Journal of Chinese Integrative Medicine	N	Y	Y	Y	N	Y	N	N	N
Intensive training, physiotherapy, or manipulation for patients with chronic neck pain. A prospective, single-blinded, randomized clinical trial	Jordan A	1998	Spine	Y	Y	Y	Y	N	Y	Y	Y	Y
Clinical analysis of Tuina manipulation treating in 146 cases of lumbar disc herniation	Ju YD	2014	China Foreign Med Treat	N	N	N	N	N	N	N	Y	N
A randomized controlled trial of exercise and manipulative therapy for cervicogenic headache	Jull G	2002	Spine	Y	Y	Y	Y	N	Y	Y	N	Y
A randomised controlled trial of spinal manipulative therapy in acute low back pain	Jüni P	2009	Annals of the Rheumatic Diseases	Y	Y	Y	Y	N	Y	Y	Y	Y
Efficacy of lumbar mobilization on postpartum low back pain in Egyptian females: A randomized control trial	Kamel DM	2016	Journal of Back and Musculoskeletal Rehabilitation	Y	Y	Y	Y	N	Y	Y	N	N
The immediate effects of mobilization technique on pain and range of motion in patients presenting with unilateral neck pain: a randomized controlled trial	Kanlayanaphontporn R	2009	Archives of Physical Medicine and Rehabilitation	Y	Y	Y	Y	N	Y	Y	Y	Y
Immediate effects of the central posteroanterior mobilization technique on pain and range of motion in patients with mechanical neck pain	Kanlayanaphontporn R	2010	Disability and Rehabilitation	Y	Y	Y	Y	N	Y	Y	Y	Y
Thoracic Spine Manipulation in Individuals With Subacromial Impingement Syndrome Does Not Immediately Alter Thoracic Spine Kinematics, Thoracic Excursion, or Scapular Kinematics: A Randomized Controlled Trial	Kardouni JR	2015	Journal of Orthopaedic and Sports Physical Therapy	Y	Y	Y	Y	N	Y	Y	Y	N
Immediate changes in pressure pain sensitivity after thoracic spinal manipulative therapy in patients with subacromial impingement syndrome: A randomized controlled study	Kardouni JR	2015	Manual Therapy	Y	Y	Y	Y	N	Y	Y	Y	N
Postural and symptomatic improvement after physiotherapy in patients with dizziness of suspected cervical origin	Karlberg M	1996	Archives of Physical Medicine and Rehabilitation	Y	Y	Y	Y	N	Y	Y	Y	Y
A true blind for subjects who receive spinal manipulation therapy	Kawchuk GN	2009	Archives of Physical Medicine and Rehabilitation	Y	Y	Y	Y	N	Y	Y	Y	N
Efficacy of C1-C2 Sustained Natural Apophyseal Glide (SNAG) Versus Posterior Anterior Vertebral Mobilization (PAVMs) in the Management of Cervicogenic Headache	Khan M	2014	Journal of Basic and Applied Sciences	N	N	N	Y	N	N	N	Y	Y
Benefits of Thoracic Thrust Manipulation when Applied with a Multi-Modal Treatment Approach in Individuals with Mechanical Neck Pain: A Pilot Randomized Trial	Khoja SS	2015	International Journal of Physical Medicine and Rehabilitation	N	N	N	Y	N	N	N	N	N
Outcome comparison among working adults with centralizing low back pain: Secondary analysis of a randomized controlled trial with 1-year follow-up	Kilpikoski S	2009	Advances in Physiotherapy	Y	Y	Y	Y	N	N	N	Y	Y
Rehabilitation with osteopathic manipulative treatment after lumbar disc surgery: A randomised, controlled pilot study	Kim BJ	2015	International Journal of Osteopathic Medicine	Y	Y	Y	Y	N	N	Y	Y	Y
Early individualised manipulative rehabilitation following lumbar open laser microdiscectomy improves early post-operative functional disability: A randomized, controlled pilot study	Kim BJ	2016	Journal of Back and Musculoskeletal Rehabilitation	Y	Y	Y	Y	N	Y	Y	Y	N
Manipulative rehabilitation applied soon after lumbar disc surgery improves late post-operative functional disability: A preliminary 2-year follow-up study	Kim BJ	2017	Journal of Back and Musculoskeletal Rehabilitation	Y	Y	Y	Y	N	Y	Y	Y	Y
The comparison of the results of manual therapy versus physiotherapy methods used in treatment of patients with low back pain syndromes	Kinalski R	1989	Manual Medicine	N	Y	Y	N	N	N	N	Y	N
Randomised trial of a brief physiotherapy intervention compared with usual physiotherapy for neck pain patients: outcomes and patients' preference	Klaber Moffett JA	2005	BMJ	Y	Y	Y	Y	N	Y	Y	Y	Y
Strain-counterstrain to treat restrictions of the mobility of the cervical spine in patients with neck pain: a sham-controlled randomized trial	Klein R	2013	Complementary Therapies in Medicine	Y	Y	Y	Y	N	Y	Y	Y	Y
Improving functional ability in the elderly via the Spencer technique, an osteopathic manipulative treatment: a randomized, controlled trial	Knebl JA	2002	Journal of the American Osteopathic Association	Y	Y	Y	Y	N	Y	N	Y	N
Randomised clinical trial of manipulative therapy and physiotherapy for persistent back and neck complaints: results of one year follow up	Koes BW	1992	BMJ	N	Y	Y	Y	N	Y	Y	Y	Y
Efficacy of manual therapy and physiotherapy for back and neck complaints (Thesis)	Koes BW	1992	den Haag: Cip-Gegevens Koninklijke Bibliotheek	N	Y	N	N	N	N	N	N	N
A blinded randomized clinical trial of manual therapy and physiotherapy for chronic back and neck complaints: physical outcome measures	Koes BW	1992	Journal of Manipulative and Physiological Therapeutics	N	Y	Y	Y	Y	Y	Y	N	N
The effectiveness of manual therapy, physiotherapy, and treatment by the general practitioner for nonspecific back and neck complaints. A randomized clinical trial	Koes BW	1992	Spine	Y	Y	Y	Y	N	Y	Y	Y	N
A randomized clinical trial of manual therapy and physiotherapy for persistent back and neck complaints: subgroup analysis and relationship between outcome measures	Koes BW	1993	Journal of Manipulative and Physiological Therapeutics	N	Y	Y	Y	Y	Y	Y	Y	N
[Cervicobrachialgia. A controlled trial with conventional therapy and manipulation]	Kogstad OA	1978	Tidsskrift for den Norske Lægeforening	N	Y	Y	N	N	Y	N	Y	N
The effect of spinal manipulation on pain and prostaglandin levels in women with primary dysmenorrhea	Kokjohn K	1992	Journal of Manipulative and Physiological Therapeutics	N	Y	Y	Y	Y	Y	Y	Y	N
Economic evaluation of four treatments for low-back pain: results from a randomized controlled trial	Kominski GF	2005	Medical Care	Y	Y	Y	Y	N	Y	Y	Y	Y
Flexion mobilizations with movement techniques: the immediate effects on range of movement and pain in subjects with low back pain	Konstantinou K	2007	Journal of Manipulative and Physiological Therapeutics	Y	Y	Y	Y	Y	Y	Y	Y	N
Cost effectiveness of physiotherapy, manual therapy, and general practitioner care for neck pain: economic evaluation alongside a randomised controlled trial	Korthals-de Bos IB	2003	BMJ	Y	Y	Y	Y	N	Y	Y	Y	Y
The immediate effects of upper thoracic translatoric spinal manipulation on cervical pain and range of motion: a randomized clinical trial	Krauss J	2008	Journal of Manual and Manipulative Therapy	Y	Y	Y	Y	N	Y	N	Y	Y
Spinal mobilization vs conventional physiotherapy in the management of chronic low back pain due to spinal disk degeneration: a randomized controlled trial	Krekoukias G	2017	Journal of Manual and Manipulative Therapy	Y	Y	Y	Y	N	Y	Y	Y	Y
The effectiveness of thoracic manipulation on patients with chronic mechanical neck pain—a randomized controlled trial	Lau HM	2011	Manual Therapy	Y	Y	Y	Y	N	Y	Y	N	N
Effects of spinal manipulation on trunk proprioception in subjects with chronic low back pain during symptom remission	Learman KE	2009	Journal of Manipulative and Physiological Therapeutics	Y	Y	Y	Y	Y	Y	Y	Y	Y
Thrust and nonthrust manipulation for older adults with low back pain: an evaluation of pain and disability	Learman KE	2013	Journal of Manipulative and Physiological Therapeutics	Y	Y	Y	Y	Y	Y	Y	Y	N
A randomized controlled trial comparing manipulation with mobilization for recent onset neck pain	Leaver AM	2010	Archives of Physical Medicine and Rehabilitation	Y	Y	Y	Y	N	Y	Y	Y	Y
A Comparison of the Effects of PNF, ESWT, and TPI on Pain and Function of Patients with Myofascial Pain Syndrome	Lee JH	2013	Journal of Physical Therapy Science	N	Y	N	Y	N	N	Y	Y	N
Effect of thoracic manipulation and deep craniocervical flexor training on pain, mobility, strength, and disability of the neck of patients with chronic nonspecific neck pain: a randomized clinical trial	Lee KW	2016	Journal of Physical Therapy Science	N	Y	N	Y	N	Y	Y	Y	Y
Exploring patient satisfaction: a secondary analysis of a randomized clinical trial of spinal manipulation, home exercise, and medication for acute and subacute neck pain	Leininger BD	2014	Journal of Manipulative and Physiological Therapeutics	Y	Y	Y	Y	Y	Y	Y	Y	N
A randomized clinical trial comparing two physiotherapy interventions for chronic low back pain	Lewis JS	2005	Spine	Y	Y	Y	Y	N	Y	Y	Y	N
[Comparative study on effects of manipulation treatment and transcutaneous electrical nerve stimulation on patients with cervicogenic headache]	Li C	2007	Journal of Chinese Integrative Medicine	N	Y	Y	Y	N	Y	N	Y	N
Tuina manipulation in treating lumbar disc herniation	Li YJ	2013	China Med Guide	N	N	N	N	N	N	N	Y	Y
Patient satisfaction and clinical outcomes associated with osteopathic manipulative treatment	Licciardone JC	2002	Journal of the American Osteopathic Association	Y	N	Y	Y	N	Y	N	Y	N
Osteopathic manipulative treatment for chronic low back pain: a randomized controlled trial	Licciardone JC	2003	Spine	Y	Y	Y	Y	N	Y	Y	Y	N
A randomized controlled trial of osteopathic manipulative treatment following knee or hip arthroplasty	Licciardone JC	2004	Journal of the American Osteopathic Association	Y	Y	Y	Y	N	Y	N	Y	Y
Osteopathic manipulative treatment of back pain and related symptoms during pregnancy: a randomized controlled trial	Licciardone JC	2010	American Journal of Obstetrics and Gynecology	Y	Y	Y	Y	N	Y	Y	Y	Y
Associations of cytokine concentrations with key osteopathic lesions and clinical outcomes in patients with nonspecific chronic low back pain: results from the OSTEOPATHIC Trial	Licciardone JC	2012	Journal of the American Osteopathic Association	Y	Y	Y	Y	N	Y	Y	Y	Y
Osteopathic manual treatment and ultrasound therapy for chronic low back pain: a randomized controlled trial	Licciardone JC	2013	Annals of Family Medicine	Y	Y	N	Y	N	Y	Y	Y	Y
Osteopathic manual treatment in patients with diabetes mellitus and comorbid chronic low back pain: subgroup results from the OSTEOPATHIC Trial	Licciardone JC	2013	Journal of the American Osteopathic Association	Y	Y	Y	Y	N	Y	N	Y	Y
Outcomes of osteopathic manual treatment for chronic low back pain according to baseline pain severity: results from the OSTEOPATHIC Trial	Licciardone JC	2013	Manual Therapy	Y	Y	Y	Y	N	Y	Y	Y	N
The effectiveness of Long's manipulation on patients with chronic mechanical neck pain: a randomized controlled trial	Lin JH	2013	Manual Therapy	Y	Y	Y	Y	N	Y	Y	Y	N
Treatment of protrusion of lumbar intervertebral disc by pulling and turning manipulations	Liu J	2000	Journal of Traditional Chinese Medicine	N	Y	Y	Y	N	Y	N	Y	Y
Analysis of pressing from JingLuo in treating lumbar disc herniation	Liu W	2012	Clin J Chin Med	N	N	N	N	N	N	N	Y	N
Immediate effects of active cranio-cervical flexion exercise versus passive mobilisation of the upper cervical spine on pain and performance on the cranio-cervical flexion test	Lluch E	2014	Manual Therapy	Y	Y	Y	Y	N	Y	Y	N	N
Mobilization versus manipulations versus sustain apophyseal natural glide techniques and interaction with psychological factors for patients with chronic neck pain: randomized controlled trial	Lopez-Lopez A	2015	European Journal of Physical and Rehabilitation Medicine	Y	Y	Y	Y	N	Y	Y	N	Y
An open controlled assessment of osteopathic manipulation in nonspecific low-back pain	MacDonald RS	1990	Spine	Y	Y	Y	Y	N	Y	Y	Y	Y
Effects of traditional Thai massage versus joint mobilization on substance P and pain perception in patients with non-specific low back pain	Mackawan S	2007	Journal of Bodywork and Movement Therapies	Y	Y	Y	Y	N	N	N	Y	Y
Joint mobilization vs massage for chronic mechanical neck pain: a pilot study to assess recruitment strategies and estimate outcome measure variability	Madson TJ	2010	Journal of Manipulative and Physiological Therapeutics	Y	Y	Y	Y	Y	Y	Y	Y	N
A randomised controlled trial on the effectiveness of osteopathic manipulative treatment of chronic low back pain	Mandara A	2008	International Journal of Osteopathic Medicine	Y	Y	N	Y	N	N	N	Y	Y
A randomised controlled trial of post-operative rehabilitation after surgical decompression of the lumbar spine	Mannion AF	2007	European Spine Journal	N	Y	Y	Y	N	Y	Y	Y	N
Efficacy of cervical spine mobilization versus peripheral nerve slider techniques in cervicobrachial pain syndrome: A randomized clinical trial	Marks M	2011	Journal of Physical Therapy	Y	Y	Y	Y	N	N	N	Y	Y
A randomised controlled trial of preventive spinal manipulation with and without a home exercise program for patients with chronic neck pain	Martel J	2011	BMC Musculoskeletal Disorders	Y	Y	Y	Y	Y	Y	Y	Y	Y
Immediate effects on neck pain and active range of motion after a single cervical high-velocity low-amplitude manipulation in subjects presenting with mechanical neck pain: a randomized controlled trial	Martínez-Segura R	2006	Journal of Manipulative and Physiological Therapeutics	Y	Y	Y	Y	Y	Y	Y	Y	Y
Immediate changes in widespread pressure pain sensitivity, neck pain, and cervical range of motion after cervical or thoracic thrust manipulation in patients with bilateral chronic mechanical neck pain: a randomized clinical trial	Martínez-Segura R	2012	Journal of Orthopaedic and Sports Physical Therapy	Y	Y	Y	Y	N	Y	Y	Y	Y
Short-term combined effects of thoracic spine thrust manipulation and cervical spine nonthrust manipulation in individuals with mechanical neck pain: a randomized clinical trial	Masaracchio M	2013	Journal of Orthopaedic and Sports Physical Therapy	Y	Y	Y	Y	N	Y	Y	N	N
Back pain and sciatica: controlled trials of manipulation, traction, sclerosant and epidural injections	Mathews JA	1987	British Journal of Rheumatology	N	Y	Y	Y	N	Y	Y	Y	N
Manipulation and traction for lumbago and sciatica: Physiotherapeutic techniques used in two controlled trials	Mathews W	1988	Physiotherapy Practice	Y	Y	Y	Y	N	N	N	Y	Y
The role of physiotherapy in the management of acute neck sprains following road-traffic accidents	McKinney LA	1989	Archives of Emergency Medicine	N	Y	Y	Y	N	Y	Y	Y	Y
Manipulation or microdiskectomy for sciatica? A prospective randomized clinical study	McMorland G	2010	Journal of Manipulative and Physiological Therapeutics	Y	Y	Y	Y	Y	Y	Y	Y	Y
Intramuscular ketorolac versus osteopathic manipulative treatment in the management of acute neck pain in the emergency department: a randomized clinical trial	McReynolds TM	2005	Journal of the American Osteopathic Association	Y	Y	Y	Y	N	Y	N	Y	Y
Low back pain of mechanical origin: randomised comparison of chiropractic and hospital outpatient treatment	Meade TW	1990	BMJ	Y	Y	Y	Y	Y	Y	Y	N	N
Randomised comparison of chiropractic and hospital outpatient management for low back pain: results from extended follow up	Meade TW	1995	BMJ	Y	Y	Y	Y	N	Y	Y	Y	Y
Early mobilization of acute whiplash injuries	Mealy K	1986	British Medical Journal	N	Y	N	Y	N	Y	Y	Y	N
Randomized, controlled trial of breath therapy for patients with chronic low-back pain	Mehling WE	2005	Alternative Therapies in Health and Medicine	Y	Y	Y	Y	N	Y	Y	Y	N
Osteopathic manipulation treatment versus therapeutic exercises in patients with chronic nonspecific low back pain: A randomized, controlled and double-blind study	Meirelles FO	2020	Journal of Back and Musculoskeletal Rehabilitation	Y	Y	Y	Y	N	Y	N	Y	Y
Validation of a sham comparator for thoracic spinal manipulation in patients with shoulder pain	Michener LA	2015	Manual Therapy	N	Y	Y	Y	N	Y	Y	Y	Y
A prospective randomised controlled trial of spinal manipulation and ultrasound in the treatment of chronic low back pain	Mohseni-Bandpei MA	2006	Physiotherapy	Y	Y	Y	Y	N	N	Y	Y	Y
Changes in pain perception after pelvis manipulation in women with primary dysmenorrhea: a randomized controlled trial	Molins-Cubero S	2014	Pain Medicine	Y	Y	Y	Y	N	Y	Y	Y	Y
Manipulative therapy in the treatment of benign cervicobrachialgia of mechanical origin	Moretti B	2004	La Chirurgia Degli Organi di Movimento	N	Y	Y	Y	N	Y	N	Y	Y
Manipulation in the Treatment of Acute Low Back Pain	Morton JE	1999	Journal of Manual and Manipulative Therapy	Y	Y	Y	Y	N	N	N	Y	Y
Combined physiotherapy and education is efficacious for chronic low back pain	Moseley L	2002	Australian Journal of Physiotherapy	Y	Y	Y	Y	N	Y	Y	Y	Y
Long-term follow-up of a randomized clinical trial assessing the efficacy of medication, acupuncture, and spinal manipulation for chronic mechanical spinal pain syndromes	Muller R	2005	Journal of Manipulative and Physiological Therapeutics	Y	Y	Y	Y	Y	Y	Y	Y	Y
The effect of spinal manipulation on the efficacy of a rehabilitation protocol for patients with chronic neck pain: a pilot study	Murphy B	2010	Journal of Manipulative and Physiological Therapeutics	Y	Y	Y	Y	Y	Y	Y	Y	Y
Effect of slump stretching versus lumbar mobilization with exercise in subjects with non-radicular low back pain: a randomized clinical trial	Nagrale AV	2012	Journal of Manual and Manipulative Therapy	Y	Y	Y	Y	N	Y	N	Y	Y
Neural tissue management provides immediate clinically relevant benefits without harmful effects for patients with nerve-related neck and arm pain: a randomised trial	Nee RJ	2012	Journal of Physiotherapy	Y	Y	Y	Y	N	Y	Y	Y	Y
The efficacy of spinal manipulation, amitriptyline and the combination of both therapies for the prophylaxis of migraine headache	Nelson CF	1998	Journal of Manipulative and Physiological Therapeutics	Y	Y	Y	Y	Y	Y	Y	Y	Y
Chronic asthma and chiropractic spinal manipulation: a randomized clinical trial	Nielsen NH	1995	Clinical and Experimental Allergy	N	Y	Y	Y	N	Y	Y	Y	N
A randomized trial of combined manipulation, stabilizing exercises, and physician consultation compared to physician consultation alone for chronic low back pain	Niemistö L	2003	Spine	Y	Y	Y	Y	N	Y	Y	N	Y
Predictive factors for 1-year outcome of chronic low back pain following manipulation, stabilizing exercises, and physician consultation or physician consultation alone	Niemistö L	2004	Journal of Rehabilitation Medicine	Y	Y	Y	Y	N	Y	Y	Y	Y
Cost-effectiveness of combined manipulation, stabilizing exercises, and physician consultation compared to physician consultation alone for chronic low back pain: a prospective randomized trial with 2-year follow-up	Niemistö L	2005	Spine	Y	Y	Y	Y	N	Y	Y	Y	N
A randomized controlled trial of the effect of spinal manipulation in the treatment of cervicogenic headache	Nilsson N	1995	Journal of Manipulative and Physiological Therapeutics	Y	Y	Y	Y	Y	Y	Y	Y	N
Lasting changes in passive range motion after spinal manipulation: a randomized, blind, controlled trial	Nilsson N	1996	Journal of Manipulative and Physiological Therapeutics	Y	Y	Y	Y	Y	Y	N	Y	N
The effect of spinal manipulation in the treatment of cervicogenic headache	Nilsson N	1997	Journal of Manipulative and Physiological Therapeutics	Y	Y	Y	Y	Y	Y	N	Y	Y
Effect of oblique pulling manipulation on lumbar disc herniation [Dissertation]	Ning GL	2015	Zhengzhou, China: Henan University of Chinese Medicine	N	N	N	N	N	N	N	Y	Y
Treatment of acute cervical pain–a comparative group study	Nordemar R	1981	Pain	Y	Y	Y	Y	N	Y	Y	Y	Y
Relative therapeutic efficacy of vertebral manipulation and conventional treatment in back pain management	Nwuga VC	1982	American Journal of Physical Medicine	Y	Y	Y	Y	N	Y	Y	Y	N
A new approach to the treatment of chronic low back pain	Ongley MJ	1987	Lancet	Y	Y	Y	Y	N	Y	Y	Y	N
Orthopaedic manual therapy, McKenzie method or advice only for low back pain in working adults: a randomized controlled trial with one year follow-up	Paatelma M	2008	Journal of Rehabilitation Medicine	Y	Y	Y	Y	N	Y	Y	Y	Y
Improvement after chiropractic care in cervicocephalic kinesthetic sensibility and subjective pain intensity in patients with nontraumatic chronic neck pain	Palmgren PJ	2006	Journal of Manipulative and Physiological Therapeutics	Y	Y	Y	Y	Y	Y	Y	Y	N
Massage and modality effects on treatment of sub-acute and chronic non specific low back pain	Panahi F	2011	Behbood J	N	N	N	Y	N	N	N	Y	N
Effects of resistance training and chiropractic treatment in women with fibromyalgia	Panton LB	2009	Journal of Alternative and Complementary Medicine	Y	Y	Y	Y	N	Y	Y	Y	Y
Impact of osteopathic therapy on proprioceptive balance and quality of life in patients with dizziness	Papa L	2017	Journal of Bodywork and Movement Therapies	Y	Y	Y	Y	N	Y	Y	Y	Y
A controlled trial of cervical manipulation of migraine	Parker GB	1978	Australian and New Zealand Journal of Medicine	N	Y	Y	Y	N	Y	Y	Y	Y
Why does migraine improve during a clinical trial? Further results from a trial of cervical manipulation for migraine	Parker GB	1980	Australian and New Zealand Journal of Medicine	N	Y	Y	Y	N	Y	N	Y	Y
Efficacy of osteopathic manipulative treatment for low back pain in euhydrated and hypohydrated conditions: a randomized crossover trial	Parker J	2012	Journal of the American Osteopathic Association	Y	Y	Y	Y	N	Y	N	Y	Y
A clinical trial investigating the effect of two manipulative approaches in the treatment of mechanical neck pain: a pilot study	Parkin-Smith GF	1998	Journal of the Neuromusculoskeletal System	Y	Y	N	N	N	N	Y	Y	Y
Effects of different treatments on postural performance in patients with cervical root compression. A randomized prospective study assessing the importance of the neck in postural control	Persson LC	1996	Journal of Vestibular Research	N	Y	Y	Y	N	Y	Y	Y	Y
Cervical radiculopathy: pain, muscle weakness and sensory loss in patients with cervical radiculopathy treated with surgery, physiotherapy or cervical collar. A prospective, controlled study	Persson LC	1997	European Spine Journal	N	Y	Y	Y	N	Y	N	N	N
Long-lasting cervical radicular pain managed with surgery, physiotherapy, or a cervical collar. A prospective, randomized study	Persson LC	1997	Spine	Y	Y	Y	Y	N	Y	Y	Y	N
Pain, coping, emotional state and physical function in patients with chronic radicular neck pain. A comparison between patients treated with surgery, physiotherapy or neck collar–a blinded, prospective randomized study	Persson LC	2001	Disability and Rehabilitation	Y	Y	Y	Y	N	Y	Y	Y	N
The McKenzie method compared with manipulation when used adjunctive to information and advice in low back pain patients presenting with centralization or peripheralization: a randomized controlled trial	Petersen T	2011	Spine	Y	Y	Y	Y	N	Y	Y	Y	Y
A pilot randomized controlled trial comparing the efficacy of exercise, spinal manipulation, and neuro emotional technique for the treatment of pregnancy-related low back pain	Peterson CD	2012	Chiropractic and Manual Therapies	Y	Y	Y	Y	Y	Y	N	Y	Y
The effects of spinal manipulation on the intensity of emotional arousal in phobic subjects exposed to a threat stimulus: a randomized, controlled, double-blind clinical trial	Peterson KB	1997	Journal of Manipulative and Physiological Therapeutics	Y	Y	Y	Y	Y	Y	Y	Y	Y
Use of spinal manipulative therapy in the treatment of duodenal ulcer: a pilot study	Pikalov AA	1994	Journal of Manipulative and Physiological Therapeutics	N	N	Y	Y	Y	Y	Y	N	N
Immediate and Short-Term Effects of Upper Thoracic Manipulation on Myoelectric Activity of Sternocleidomastoid Muscles in Young Women With Chronic Neck Pain: A Randomized Blind Clinical Trial	Pires PF	2015	Journal of Manipulative and Physiological Therapeutics	Y	Y	Y	Y	Y	Y	Y	Y	Y
Is a behavioral graded activity program more effective than manual therapy in patients with subacute neck pain? Results of a randomized clinical trial	Pool JJ	2010	Spine	Y	Y	Y	Y	N	Y	Y	Y	Y
A prospective randomized three-week trial of spinal manipulation, transcutaneous muscle stimulation, massage and corset in the treatment of subacute low back pain	Pope MH	1994	Spine	Y	Y	Y	Y	N	Y	Y	Y	N
Efficacy of various forms of conservative treatment in low back pain: a comparative study	Postacchini F	1988	Neuro-Orthopedics	N	Y	Y	N	Y	N	N	Y	N
Multimodal treatment to prevent the late whiplash syndrome	Provinciali L	1996	Scandinavian Journal of Rehabilitation Medicine	Y	Y	Y	Y	N	Y	Y	Y	N
Thoracic spine thrust manipulation versus cervical spine thrust manipulation in patients with acute neck pain: a randomized clinical trial	Puentedura EJ	2011	Journal of Orthopaedic and Sports Physical Therapy	Y	Y	Y	Y	N	Y	Y	N	Y
Irritable bowel syndrome treated by traditional Chinese spinal orthopedic manipulation	Qu L	2012	Journal of Traditional Chinese Medicine	N	Y	Y	Y	N	Y	Y	Y	N
Parkinsonian gait ameliorated with a moving handrail, not with a banister	Rabin E	2015	Archives of Physical Medicine and Rehabilitation	Y	Y	Y	Y	N	Y	Y	Y	N
A randomized trial comparing manual physical therapy to therapeutic exercises, to a combination of therapies, for the treatment of cervical radiculopathy	Ragonese J	2009	Orthopaedic Practice	Y	Y	N	N	N	N	N	Y	Y
Manipulation in the treatment of low back pain: a randomized clinical trial	Rasmussen GG	1979	Manuelle Medizin	N	Y	Y	N	N	N	N	Y	Y
Manipulation does not add to the effect of extension exercises in chronic low-back pain (LBP). A randomized, controlled, double blind study	Rasmussen J	2008	Joint Bone Spine	N	Y	Y	Y	N	Y	Y	Y	N
Stabilizing training compared with manual treatment in sub-acute and chronic low-back pain	Rasmussen-Barr E	2003	Manual Therapy	Y	Y	Y	Y	N	Y	Y	Y	Y
A comparison of spinal manipulation with physical therapy for treatment of patients with mechanical low back pain	Rayegani SM	2002	J. Babol Univ. Med. Sci	N	N	N	Y	N	N	N	Y	N
Efficiency of manual therapy on patients with cervicogenic headache: a randomized single blinded controlled trial	Reginiussen T	2000	International Federation of Manipulation Therapy	N	Y	N	Y	N	N	N	N	Y
Psychosocial differences as predictors for recovery from chronic low back pain following manipulation, stabilizing exercises and physician consultation or physician consultation alone	Riipinen M	2005	Journal of Rehabilitation Medicine	Y	Y	Y	Y	N	Y	Y	N	Y
Short-term effects of thoracic spinal manipulations and message conveyed by clinicians to patients with musculoskeletal shoulder symptoms: a randomized clinical trial	Riley SP	2015	Journal of Manual and Manipulative Therapy	Y	Y	Y	Y	N	Y	N	Y	N
Osteopathische Behandlung bei Frauen mit Blasenentleerungsstörungen	Ringkamp C	2009	AFO, Germany	N	N	N	N	N	N	N	Y	Y
Dynamic surface electromyographic responses in chronic low back pain treated by traditional bone setting and conventional physical therapy	Ritvanen T	2007	Journal of Manipulative and Physiological Therapeutics	Y	Y	Y	Y	Y	Y	Y	Y	Y
Chiropractic adjustments: results of a controlled clinical trial in Egypt	Rupert RL	1985	International Review of Chiropractic	N	Y	N	N	Y	N	N	Y	N
Short-term effects of kinesio taping versus cervical thrust manipulation in patients with mechanical neck pain: a randomized clinical trial	Saavedra-Hernández M	2012	Journal of Orthopaedic and Sports Physical Therapy	Y	Y	Y	Y	N	Y	Y	Y	Y
Short-term effects of spinal thrust joint manipulation in patients with chronic neck pain: a randomized clinical trial	Saavedra-Hernández M	2013	Clinical Rehabilitation	Y	Y	Y	Y	N	Y	Y	Y	Y
Chiropractic manipulative therapy and low-level laser therapy in the management of cervical facet dysfunction: a randomized controlled study	Saayman L	2011	Journal of Manipulative and Physiological Therapeutics	Y	Y	Y	Y	Y	Y	Y	N	N
Immediate changes in neck pain intensity and widespread pressure pain sensitivity in patients with bilateral chronic mechanical neck pain: a randomized controlled trial of thoracic thrust manipulation vs non-thrust mobilization	Salom-Moreno J	2014	Journal of Manipulative and Physiological Therapeutics	Y	Y	Y	Y	Y	Y	Y	N	Y
Mulligan versus Maitland Mobilizations In Patients with Chronic Low Back Dysfunction	Samir SM	2016	International Journal of PharmTech Research	N	Y	Y	Y	N	N	N	Y	N
Chiropractic adjustive manipulation on subjects with acute low back pain: visual analog pain scores and plasma beta-endorphin levels	Sanders GE	1990	Journal of Manipulative and Physiological Therapeutics	N	Y	Y	Y	Y	Y	Y	Y	N
Chiropractic manipulation in the treatment of acute back pain and sciatica with disc protrusion: a randomized double-blind clinical trial of active and simulated spinal manipulations	Santilli V	2006	The Spine Journal	N	Y	Y	Y	N	Y	N	Y	N
Active Visceral Manipulation Associated With Conventional Physiotherapy in People With Chronic Low Back Pain and Visceral Dysfunction: A Preliminary, Randomized, Controlled, Double-Blind Clinical Trial	Santos LV	2019	Journal of Chiropractic Medicine	Y	Y	N	Y	Y	Y	Y	Y	N
Effect of spinal manipulation on postural instability in patients with non specific low back pain	Sarker KK	2017	International Journal of Pharma and Bio Sciences	N	N	N	N	N	N	N	Y	Y
Active or passive treatment for neck-shoulder pain in occupational health care? A randomized controlled trial	Savolainen A	2004	Occupational Medicine	N	Y	Y	Y	N	Y	Y	N	N
The effect of an analgesic mobilization technique when applied at symptomatic or asymptomatic levels of the cervical spine in subjects with neck pain: a randomized controlled trial	Schomacher J	2009	Journal of Manual and Manipulative Therapy	Y	Y	Y	Y	N	Y	N	N	N
Osteopathic treatment of patients with chronic non-specific neck pain: a randomised controlled trial of efficacy	Schwerla F	2008	Forschende Komplementärmedizin	N	Y	Y	Y	N	Y	Y	Y	Y
Osteopathic treatment of women with persistent low back/pelvic girdle pain postpartum	Schwerla F	2012	Akademie für Osteopathie	N	N	N	N	N	N	N	Y	N
Osteopathic Manipulative Therapy in Women With Postpartum Low Back Pain and Disability: A Pragmatic Randomized Controlled Trial	Schwerla F	2015	Journal of the American Osteopathic Association	Y	Y	Y	Y	N	Y	N	N	N
Randomized controlled trial of neural mobilization after spinal surgery	Scrimshaw SV	2001	Spine	N	Y	Y	Y	N	Y	Y	Y	Y
Conservative treatment in patients sick-listed for acute low-back pain: a prospective randomised study with 12 months' follow-up	Seferlis T	1998	European Spine Journal	N	Y	Y	Y	N	Y	N	Y	N
Cost-minimisation analysis of three conservative treatment programmes in 180 patients sick-listed for acute low-back pain	Seferlis T	2000	Scandinavian Journal of Primary Health Care	N	Y	Y	Y	N	Y	Y	Y	Y
Does maintained spinal manipulation therapy for chronic nonspecific low back pain result in better long-term outcome?	Senna MK	2011	Spine	Y	Y	Y	Y	N	Y	Y	Y	N
A randomized clinical trial of manual versus mechanical force manipulation in the treatment of sacroiliac joint syndrome	Shearer KA	2005	Journal of Manipulative and Physiological Therapeutics	Y	Y	Y	Y	Y	Y	Y	Y	Y
Randomized trial of therapeutic massage for chronic neck pain	Sherman KJ	2009	The Clinical Journal of Pain	Y	Y	Y	Y	N	Y	Y	Y	Y
Comparison between the effects of Chuna manipulation therapy and cervical traction treatment on pain in patients with herniated cervical disc: a randomized clinical pilot trial	Shin BC	2006	The American Journal of Chinese Medicine	N	Y	Y	N	N	Y	Y	Y	N
The effect of sustained natural apophyseal glides on headache, duration and cervical function in women with cervicogenic headache	Shin E-J	2014	Journal of Exercise Rehabilitation	N	N	N	Y	N	Y	N	Y	Y
Manipulation of the lumbar spine with the patient under general anesthesia: evaluation by electromyography and clinical-neurologic examination of its use for lumbar nerve root compression syndrome	Siehl D	1971	Journal of the American Osteopathic Association	N	Y	Y	N	N	Y	N	Y	N
Immediate effects of a thoracic spine thrust manipulation on the autonomic nervous system: a randomized clinical trial	Sillevis R	2010	Journal of Manual and Manipulative Therapy	Y	Y	Y	Y	N	Y	N	Y	Y
Immediate effects of the audible pop from a thoracic spine thrust manipulation on the autonomic nervous system and pain: a secondary analysis of a randomized clinical trial	Sillevis R	2011	Journal of Manipulative and Physiological Therapeutics	Y	Y	Y	Y	Y	Y	Y	N	N
Controlled trial of mobilisation and manipulation for patients with low back pain in general practice	Sims-Williams H	1978	British Medical Journal	N	Y	Y	Y	N	Y	Y	Y	N
Controlled trial of mobilisation and manipulation for low back pain: hospital patients	Sims-Williams H	1979	British Medical Journal	N	Y	Y	Y	N	Y	Y	Y	N
Cost and effectiveness analysis of chiropractic and physiotherapy treatment for low back and neck pain. Six-month follow-up	Skargren EI	1997	Spine	Y	Y	Y	Y	N	Y	Y	Y	Y
Predictive factors for 1-year outcome of low-back and neck pain in patients treated in primary care: comparison between the treatment strategies chiropractic and physiotherapy	Skargren EI	1998	Pain	Y	Y	Y	Y	N	Y	Y	N	N
One-year follow-up comparison of the cost and effectiveness of chiropractic and physiotherapy as primary management for back pain. Subgroup analysis, recurrence, and additional health care utilization	Skargren EI	1998	Spine	Y	Y	Y	Y	N	Y	Y	Y	Y
Naprapathic manual therapy or evidence-based care for back and neck pain: a randomized, controlled trial	Skillgate E	2007	The Clinical Journal of Pain	Y	Y	Y	Y	N	Y	Y	Y	Y
The long-term effects of naprapathic manual therapy on back and neck pain—results from a pragmatic randomized controlled trial	Skillgate E	2010	BMC Musculoskeletal Disorders	Y	Y	Y	Y	N	Y	Y	Y	Y
Manipulation for chronic neck pain. A double-blind controlled study	Sloop PR	1982	Spine	N	Y	Y	Y	N	Y	Y	Y	N
Dose optimization for spinal treatment effectiveness: a randomized controlled trial investigating the effects of high and low mobilization forces in patients with neck pain	Snodgrass SJ	2014	Journal of Orthopaedic and Sports Physical Therapy	Y	Y	Y	Y	N	Y	Y	Y	Y
Evaluation of the Toftness system of chiropractic adjusting for subjects with chronic back pain, chronic tension headaches, or primary dysmenorrhea	Snyder BJ	1996	Chiropractic Techniques	Y	Y	N	N	N	N	N	Y	Y
Cervical lateral glide increases nociceptive flexion reflex threshold but not pressure or thermal pain thresholds in chronic whiplash associated disorders: A pilot randomised controlled trial	Sterling M	2010	Manual Therapy	Y	Y	Y	Y	N	Y	Y	Y	N
A feasibility study assessing manual therapies to different regions of the spine for patients with subacute or chronic neck pain	Strunk RG	2008	Journal of Chiropractic Medicine	Y	Y	N	Y	Y	Y	N	N	Y
[Clinical observation of the therapeutic effect of spine fine adjusting in treating cervical spondylotic radiculopathy and the conversion of cervical curvature]	Sun WQ	2006	China Journal of Traditional Chinese Medicine and Pharmacy	N	N	N	Y	N	N	N	N	Y
Comparison of short-term response to two spinal manipulation techniques for patients with low back pain in a military beneficiary population	Sutlive TG	2009	Military Medicine	Y	Y	Y	Y	N	Y	Y	Y	N
The effects of thoracic manipulation versus mobilization for chronic neck pain: a randomized controlled trial pilot study	Suvarnnato T	2013	Journal of Physical Therapy Science	N	Y	N	Y	N	Y	Y	Y	N
Preventive spinal manipulation for patient with chronic neck pain	Thistle S	2011	Dynamic Chiropractic	Y	Y	N	N	N	N	N	Y	N
Effectiveness of spinal manipulative therapy in treatment of primary dysmenorrhea: A pilot study	Thomason PR	1979	Journal of Manipulative and Physiological Therapeutics	N	Y	N	N	Y	N	N	Y	Y
A randomized-control study of active and passive treatments for chronic low back pain following L5 laminectomy	Timm KE	1994	Journal of Orthopaedic and Sports Physical Therapy	Y	Y	Y	Y	N	Y	Y	Y	N
Musculoskeletal manipulation in the treatment of low back pain	Tobis JS	1983	Bulletin of the New York Academy of Medicine	N	Y	Y	Y	N	Y	Y	Y	N
Immediate effects of High-Velocity thrust to the cervical spine on Pressure Pain Threshold and Pain-Free Grip Strength in subjects with Lateral Epicondylalgia [Master Thesis]	Treacher A	2011	Unitec Institute of Technology	N	N	N	Y	N	N	N	Y	Y
A randomized controlled clinical trial of manipulation for chronic low back pain patients	Triano JJ	1994	Journal of Manipulative and Physiological Therapeutics	N	Y	N	N	N	N	N	Y	Y
Manipulative therapy versus education programs in chronic low back pain	Triano JJ	1995	Spine	Y	Y	Y	Y	N	Y	Y	Y	Y
Tuina manipulation in treating 60 cases of lumbar disc herniation	Tu XS	2014	YunNan Tradit Chin Med J	N	N	N	N	N	N	N	Y	Y
A randomized controlled trial of chiropractic spinal manipulative therapy for migraine	Tuchin PJ	2000	Journal of Manipulative and Physiological Therapeutics	Y	Y	Y	Y	Y	Y	Y	Y	N
A comparison of cranial electrotherapy stimulation alone or with chiropractic therapies in the treatment of fibromyalgia	Tyers S	2001	The American Chiropractor	N	N	N	N	N	N	N	Y	N
United Kingdom back pain exercise and manipulation (UK BEAM) randomised trial: effectiveness of physical treatments for back pain in primary care	UK BEAM Trial Team	2004	BMJ	Y	Y	Y	Y	N	Y	Y	Y	N
United Kingdom back pain exercise and manipulation (UK BEAM) randomised trial: cost effectiveness of physical treatments for back pain in primary care	UK BEAM Trial Team	2004	BMJ	Y	Y	Y	Y	N	Y	Y	Y	N
The effect of manual therapy and exercise in patients with chronic low back pain: Double blind randomized controlled trial	Ulger O	2017	Journal of Back and Musculoskeletal Rehabilitation	Y	Y	Y	Y	N	Y	Y	Y	N
Do baseline characteristics predict response to treatment for low back pain? Secondary analysis of the UK BEAM dataset [ISRCTN32683578]	Underwood MR	2007	Rheumatology	N	Y	Y	Y	N	Y	Y	Y	Y
A clinical trial investigating the possible effect of the supine cervical rotatory manipulation and the supine lateral break manipulation in the treatment of mechanical neck pain: a pilot study	van Schalkwyk R	2000	Journal of Manipulative and Physiological Therapeutics	Y	Y	Y	Y	Y	Y	Y	Y	N
The effect of pain reduction on perceived tension and EMG-recorded trapezius muscle activity in workers with shoulder and neck pain	Vasseljen O	1995	Scandinavian Journal of Rehabilitation Medicine	Y	Y	Y	Y	N	Y	Y	Y	N
Physical examination and self-reported pain outcomes from a randomized trial on chronic cervicogenic headache	Vavrek D	2010	Journal of Manipulative and Physiological Therapeutics	Y	Y	Y	Y	Y	Y	Y	Y	Y
Effectiveness of low back pain manipulative therapy in combination with physical therapy as compared to standard physical therapy	Venegas-Rios HL	2009	University of North Texas Health Science Center at Fort Worth	Y	Y	N	Y	N	N	N	N	N
Pain, Range of Motion and Back Strength in Chronic Mechanical Low Back Pain before and after Lumbar Mobilisation	Verma Y	2013	International Journal of Physiotherapy and Research	N	N	N	N	N	N	N	Y	N
A randomized, placebo-controlled clinical trial of chiropractic and medical prophylactic treatment of adults with tension-type headache: results from a stopped trial	Vernon H	2009	Journal of Manipulative and Physiological Therapeutics	Y	Y	Y	Y	Y	Y	Y	Y	Y
Pressure pain threshold evaluation of the effect of spinal manipulation in the treatment of chronic neck pain: a pilot study	Vernon HT	1990	Journal of Manipulative and Physiological Therapeutics	N	Y	Y	Y	Y	Y	Y	N	N
Validation of a novel sham cervical manipulation procedure	Vernon HT	2012	The Spine Journal	Y	Y	Y	Y	N	Y	Y	N	N
Osteopathic manipulative treatment in obese patients with chronic low back pain: a pilot study	Vismara L	2012	Manual Therapy	Y	Y	Y	Y	N	Y	Y	N	N
Efficacy of osteopathic manipulative treatment of female patients with migraine: results of a randomized controlled trial	Voigt K	2011	Journal of Alternative and Complementary Medicine	Y	Y	Y	Y	N	Y	Y	Y	N
Spinal high-velocity low amplitude manipulation in acute nonspecific low back pain: a double-blinded randomized controlled trial in comparison with diclofenac and placebo	von Heymann WJ	2013	Spine	Y	Y	Y	Y	N	Y	Y	N	N
Effect of treatment of temporomandibular disorders (TMD) in patients with cervicogenic headache: a single-blind, randomized controlled study	von Piekartz H	2011	Cranio: The Journal of Craniomandibular Practice	Y	Y	Y	Y	N	Y	Y	Y	Y
Short term trial of chiropractic adjustments for the relief of chronic low back pain	Waagen GN	1986	Manual Medicine	N	Y	N	N	N	N	N	Y	Y
Short-term usual chiropractic care for spinal pain: a randomized controlled trial	Walker BF	2013	Spine	Y	Y	Y	Y	N	Y	Y	Y	N
The effectiveness of manual physical therapy and exercise for mechanical neck pain: a randomized clinical trial	Walker MJ	2008	Spine	Y	Y	Y	Y	N	Y	Y	Y	N
A randomized, placebo-controlled clinical trial on the efficacy of chiropractic therapy on premenstrual syndrome	Walsh MJ	1999	Journal of Manipulative and Physiological Therapeutics	Y	Y	Y	Y	Y	Y	Y	Y	N
Early intervention for the management of acute low back pain: a single-blind randomized controlled trial of biopsychosocial education, manual therapy, and exercise	Wand BM	2004	Spine	Y	Y	Y	Y	N	Y	Y	Y	N
Tunia manipulation in treating lumbar disc herniation	Wang CE	2016	World Latest Med Inf	N	N	N	N	N	N	N	Y	Y
Clinical Research of Standard Oblique Pulling Manipulation in Treating Lumbar Disc Herniation [Dissertation]	Wang LH	2010	Changsa, China: Hunan University of Chinese Medicine	N	N	N	N	N	N	N	Y	Y
McKenzie treatment versus mulligan sustained natural apophyseal glides for chronic mechanical low back pain	Waqqar S	2016	Pakistan Journal of Medical Sciences	N	Y	Y	Y	N	Y	Y	N	Y
An open study of diflunisal, conservative and manipulative therapy in the management of acute mechanical low back pain	Waterworth RF	1985	New Zealand Medical Journal	Y	Y	Y	Y	N	Y	Y	N	N
A comparison between chiropractic management and pain clinic management for chronic low-back pain in a national health service outpatient clinic	Wilkey A	2008	Journal of Alternative and Complementary Medicine	Y	Y	Y	Y	N	Y	Y	Y	N
Randomized osteopathic manipulation study (ROMANS): pragmatic trial for spinal pain in primary care	Williams NH	2003	Family Practice	N	Y	Y	Y	N	Y	Y	Y	Y
Cost-utility analysis of osteopathy in primary care: results from a pragmatic randomized controlled trial	Williams NH	2004	Family Practice	N	Y	Y	Y	N	Y	Y	Y	Y
Comparison of physiotherapy, manipulation, and corticosteroid injection for treating shoulder complaints in general practice: randomised, single blind study	Winters JC	1997	BMJ	Y	Y	Y	Y	N	Y	N	N	N
Efficacy of chiropractic treatment on fibromyalgia syndrome: a randomized controlled trial	Wise P	2002	Eur J Chiro	N	Y	N	N	N	N	N	Y	Y
A pilot randomized clinical trial on the relative effect of instrumental (MFMA) versus manual (HVLA) manipulation in the treatment of cervical spine dysfunction	Wood TG	2001	Journal of Manipulative and Physiological Therapeutics	Y	Y	Y	Y	Y	Y	Y	Y	N
Treatment of pelvic joint dysfunction in primary care–a controlled study	Wreje U	1992	Scandinavian Journal of Primary Health Care	N	Y	Y	Y	N	Y	N	Y	N
Similar Effects of Thrust and Nonthrust Spinal Manipulation Found in Adults With Subacute and Chronic Low Back Pain: A Controlled Trial With Adaptive Allocation	Xia T	2016	Spine	Y	Y	Y	Y	N	Y	Y	Y	N
Stretching exercises vs manual therapy in treatment of chronic neck pain: a randomized, controlled cross-over trial	Ylinen J	2007	Journal of Rehabilitation Medicine	Y	Y	Y	Y	N	Y	Y	Y	Y
Manual therapy, exercise, and traction for patients with cervical radiculopathy: a randomized clinical trial	Young IA	2009	Physical Therapy	Y	Y	Y	Y	N	Y	Y	Y	N
Mobilization versus massage therapy in the treatment of cervicogenic headache: a clinical study	Youssef E	2013	Journal of Back and Musculoskeletal Rehabilitation	Y	Y	Y	Y	N	Y	Y	Y	Y
Comparison of two chiropractic techniques on pain and lateral flexion in neck pain patients: a pilot study	Yurkiw D	1996	Chiropractic Technique	Y	Y	N	N	Y	N	N	Y	Y
Effectiveness of traditional bone setting in chronic neck pain: randomized clinical trial	Zaproudina N	2007	Journal of Manipulative and Physiological Therapeutics	Y	Y	Y	Y	Y	Y	Y	Y	Y
Effectiveness of traditional bone setting in treating chronic low back pain: a randomised pilot trial	Zaproudina N	2009	Complementary Therapies in Medicine	Y	Y	Y	Y	N	Y	Y	N	N
[Observation of clinical curative effect of "oblique-pulling" maneuver in the treatment of lumbar intervertebral disc herniation]	Zhang J	2010	China Journal of Orthopaedics and Traumatology	N	Y	Y	Y	N	Y	N	N	N
[Clinical study of effect of rotational manipulation in treating cervical spondylotic radiculopathy using visual analog scales]	Zhu LG	2005	Beijing J Tradit Med	N	N	N	N	N	N	N	Y	N
[Clinical observation on rotation-traction manipulation for treatment of the cervical spondylotic of the neuro-radicular type]	Zhu LG	2005	China Journal of Orthopaedics and Traumatology	N	N	N	N	N	N	N	Y	N
[The measurement of pain and numbness in patients with cervical spondylotic radiculopathy]	Zhu LG	2009	Chinese Journal of Traditional Medical Traumatology and Orthopedics	N	N	N	N	N	N	N	Y	Y
Lumbar disc disease: comparative analysis of physical therapy treatments	Zylbergold RS	1981	Archives of Physical Medicine and Rehabilitation	N	Y	Y	Y	N	Y	Y	Y	N

*Y* yes, *N* no

## Appendix 3

See Table 10.

**Table 10:** Complete list of recall rates for all combinations of two, three and four databases

	RCTs found (*n*)	Overall recall^a^ (%)	Mean recall per SR^b^ (%)	Median recall per SR^c^ (%)	100% recall per SR^d^ (%)
*Combination of two databases*					
Cochrane Library + Google Scholar	417	94.3	95.2	100.0	83.5
Google Scholar + PEDro	416	94.1	96.4	100.0	83.5
Cochrane Library + PEDro	414	93.7	94.3	100.0	82.4
Cochrane Library + AMED	410	92.8	93.3	100.0	82.4
Cochrane Library + Index to Chiropractic Literature	409	92.5	93.2	100.0	80.0
Cochrane Library + MEDLINE/PubMed	409	92.5	93.2	100.0	78.8
Cochrane Library + EMBASE	408	92.3	93.0	100.0	78.8
CINAHL + Cochrane Library	407	92.1	92.3	100.0	77.6
Cochrane Library + Web of Science	407	92.1	92.8	100.0	77.6
EMBASE + PEDro	405	91.6	93.5	100.0	74.1
MEDLINE/PubMed + PEDro	404	91.4	93.4	100.0	72.9
Google Scholar + AMED	401	90.7	90.9	100.0	52.9
EMBASE + Google Scholar	400	90.5	92.0	100.0	61.2
Google Scholar + Index to Chiropractic Literature	399	90.3	90.5	100.0	51.8
Web of Science + PEDro	399	90.3	92.8	100.0	68.2
CINAHL + Google Scholar	398	90.0	90.0	100.0	51.8
Google Scholar + MEDLINE/PubMed	395	89.4	90.6	100.0	54.1
Google Scholar + Web of Science	394	89.1	90.7	100.0	55.3
CINAHL + EMBASE	393	88.9	90.7	100.0	60.0
CINAHL + PEDro	393	88.9	90.8	100.0	60.0
EMBASE + AMED	393	88.9	90.1	100.0	60.0
EMBASE + Index to Chiropractic Literature	391	88.5	90.2	100.0	55.3
CINAHL + MEDLINE/PubMed	390	88.2	89.3	100.0	52.9
PEDro + AMED	387	87.6	90.1	100.0	60.0
EMBASE + Web of Science	386	87.3	88.5	100.0	51.8
MEDLINE/PubMed + AMED	386	87.3	88.8	98.1	48.2
EMBASE + MEDLINE/PubMed	385	87.1	88.5	100.0	51.8
Index to Chiropractic Literature + MEDLINE/PubMed	378	85.5	86.8	94.7	43.5
Index to Chiropractic Literature + PEDro	377	85.3	88.3	98.1	49.4
MEDLINE/PubMed + Web of Science	373	84.4	86.6	93.8	42.4
CINAHL + Web of Science	369	83.5	86.4	96.8	48.2
Web of Science + AMED	356	80.5	84.2	88.9	35.3
Index to Chiropractic Literature + Web of Science	340	76.9	82.2	88.9	32.9
CINAHL + AMED	336	76.0	76.0	83.3	28.2
CINAHL + Index to Chiropractic Literature	309	69.9	69.2	75.0	23.5
Index to Chiropractic Literature + AMED	246	55.7	58.8	60.0	10.6
*Combination of three databases*					
Cochrane Library + Google Scholar + PEDro	424	95.9	97.0	100.0	90.6
Cochrane Library + Google Scholar + AMED	420	95.0	95.6	100.0	88.2
Google Scholar + PEDro + AMED	419	94.8	96.8	100.0	85.9
CINAHL + Cochrane Library + Google Scholar	418	94.6	95.3	100.0	84.7
CINAHL + Google Scholar + PEDro	418	94.6	96.4	100.0	83.5
Cochrane Library + Google Scholar + Index to Chiropractic Literature	418	94.6	95.3	100.0	84.7
Google Scholar + Index to Chiropractic Literature + PEDro	418	94.6	96.5	100.0	84.7
Cochrane Library + EMBASE + Google Scholar	417	94.3	95.2	100.0	83.5
Cochrane Library + EMBASE + PEDro	417	94.3	95.2	100.0	85.9
Cochrane Library + Google Scholar + MEDLINE/PubMed	417	94.3	95.2	100.0	83.5
Cochrane Library + Google Scholar + Web of Science	417	94.3	95.2	100.0	83.5
Cochrane Library + Index to Chiropractic Literature + PEDro	417	94.3	95.4	100.0	85.9
Cochrane Library + MEDLINE/PubMed + PEDro	417	94.3	95.2	100.0	85.9
EMBASE + Google Scholar + PEDro	417	94.3	96.4	100.0	84.7
Google Scholar + MEDLINE/PubMed + PEDro	417	94.3	96.4	100.0	84.7
Google Scholar + Web of Science + PEDro	417	94.3	96.4	100.0	84.7
Cochrane Library + PEDro + AMED	416	94.1	95.0	100.0	84.7
Cochrane Library + Web of Science + PEDro	416	94.1	95.0	100.0	84.7
CINAHL + Cochrane Library + PEDro	415	93.9	94.5	100.0	83.5
Cochrane Library + Index to Chiropractic Literature + AMED	412	93.2	93.8	100.0	84.7
Cochrane Library + MEDLINE/PubMed + AMED	412	93.2	93.5	100.0	83.5
Cochrane Library + EMBASE + AMED	411	93.0	93.4	100.0	83.5
Cochrane Library + Index to Chiropractic Literature + MEDLINE/PubMed	411	93.0	93.6	100.0	81.2
Cochrane Library + Web of Science + AMED	411	93.0	93.4	100.0	83.5
CINAHL + Cochrane Library + AMED	410	92.8	93.3	100.0	82.4
CINAHL + Cochrane Library + Index to Chiropractic Literature	410	92.8	93.4	100.0	81.2
CINAHL + Cochrane Library + MEDLINE/PubMed	410	92.8	93.2	100.0	80.0
CINAHL + EMBASE + PEDro	410	92.8	94.6	100.0	78.8
CINAHL + MEDLINE/PubMed + PEDro	410	92.8	94.6	100.0	78.8
Cochrane Library + EMBASE + Index to Chiropractic Literature	410	92.8	93.4	100.0	81.2
CINAHL + Cochrane Library + EMBASE	409	92.5	93.0	100.0	80.0
CINAHL + Cochrane Library + Web of Science	409	92.5	93.0	100.0	80.0
Cochrane Library + EMBASE + MEDLINE/PubMed	409	92.5	93.2	100.0	78.8
Cochrane Library + Index to Chiropractic Literature + Web of Science	409	92.5	93.2	100.0	80.0
Cochrane Library + MEDLINE/PubMed + Web of Science	409	92.5	93.2	100.0	78.8
EMBASE + Index to Chiropractic Literature + PEDro	409	92.5	94.6	100.0	77.6
CINAHL + EMBASE + Google Scholar	408	92.3	93.3	100.0	67.1
Cochrane Library + EMBASE + Web of Science	408	92.3	93.0	100.0	78.8
EMBASE + Google Scholar + AMED	408	92.3	93.7	100.0	70.6
EMBASE + Google Scholar + Index to Chiropractic Literature	408	92.3	93.1	100.0	64.7
EMBASE + PEDro + AMED	408	92.3	93.9	100.0	76.5
Index to Chiropractic Literature + MEDLINE/PubMed + PEDro	408	92.3	94.5	100.0	76.5
CINAHL + Web of Science + PEDro	407	92.1	94.2	100.0	76.5
MEDLINE/PubMed + PEDro + AMED	407	92.1	93.8	100.0	75.3
CINAHL + Google Scholar + AMED	406	91.9	92.0	100.0	56.5
EMBASE + Web of Science + PEDro	406	91.9	93.6	100.0	75.3
Google Scholar + Index to Chiropractic Literature + AMED	406	91.9	92.3	100.0	62.4
EMBASE + MEDLINE/PubMed + PEDro	405	91.6	93.5	100.0	74.1
Google Scholar + MEDLINE/PubMed + AMED	405	91.6	92.6	100.0	61.2
MEDLINE/PubMed + Web of Science + PEDro	405	91.6	93.5	100.0	74.1
Google Scholar + Index to Chiropractic Literature + MEDLINE/PubMed	404	91.4	92.4	100.0	60.0
Google Scholar + Web of Science + AMED	404	91.4	92.7	100.0	62.4
Index to Chiropractic Literature + Web of Science + PEDro	404	91.4	93.9	100.0	71.8
Web of Science + PEDro + AMED	404	91.4	93.6	100.0	72.9
CINAHL + Google Scholar + Index to Chiropractic Literature	403	91.2	91.4	100.0	55.3
CINAHL + Google Scholar + MEDLINE/PubMed	403	91.2	91.9	100.0	60.0
Google Scholar + Index to Chiropractic Literature + Web of Science	403	91.2	92.5	100.0	62.4
CINAHL + EMBASE + AMED	402	91.0	92.5	100.0	70.6
CINAHL + Google Scholar + Web of Science	401	90.7	91.8	100.0	60.0
EMBASE + Google Scholar + Web of Science	401	90.7	92.2	100.0	62.4
CINAHL + MEDLINE/PubMed + AMED	400	90.5	91.6	100.0	61.2
CINAHL + PEDro + AMED	400	90.5	92.4	100.0	68.2
EMBASE + Google Scholar + MEDLINE/PubMed	400	90.5	92.0	100.0	61.2
EMBASE + Index to Chiropractic Literature + AMED	399	90.3	91.8	100.0	68.2
CINAHL + EMBASE + Index to Chiropractic Literature	398	90.0	91.8	100.0	63.5
CINAHL + EMBASE + Web of Science	398	90.0	91.0	100.0	62.4
CINAHL + Index to Chiropractic Literature + PEDro	398	90.0	92.0	100.0	64.7
EMBASE + Index to Chiropractic Literature + Web of Science	398	90.0	91.0	100.0	60.0
CINAHL + EMBASE + MEDLINE/PubMed	397	89.8	91.1	100.0	61.2
CINAHL + Index to Chiropractic Literature + MEDLINE/PubMed	396	89.6	91.1	100.0	58.8
EMBASE + Index to Chiropractic Literature + MEDLINE/PubMed	396	89.6	90.8	100.0	57.6
EMBASE + MEDLINE/PubMed + AMED	396	89.6	90.6	100.0	61.2
EMBASE + Web of Science + AMED	396	89.6	90.6	100.0	62.4
Google Scholar + MEDLINE/PubMed + Web of Science	396	89.6	90.8	100.0	55.3
CINAHL + MEDLINE/PubMed + Web of Science	393	88.9	89.7	100.0	55.3
Index to Chiropractic Literature + MEDLINE/PubMed + AMED	393	88.9	91.3	100.0	60.0
Index to Chiropractic Literature + PEDro + AMED	392	88.7	91.3	100.0	64.7
EMBASE + MEDLINE/PubMed + Web of Science	388	87.8	88.8	100.0	52.9
MEDLINE/PubMed + Web of Science + AMED	388	87.8	89.2	98.4	49.4
Index to Chiropractic Literature + MEDLINE/PubMed + Web of Science	385	87.1	89.6	98.1	49.4
CINAHL + Web of Science + AMED	381	86.2	88.9	100.0	50.6
CINAHL + Index to Chiropractic Literature + Web of Science	376	85.1	88.1	100.0	50.6
Index to Chiropractic Literature + Web of Science + AMED	367	83.0	87.1	95.0	43.5
CINAHL + Index to Chiropractic Literature + AMED	344	77.8	77.7	85.7	30.6
*Combination of four databases*					
CINAHL + Cochrane Library + Google Scholar + PEDro	424	95.9	97.0	100.0	90.6
Cochrane Library + EMBASE + Google Scholar + PEDro	424	95.9	97.0	100.0	90.6
Cochrane Library + Google Scholar + Index to Chiropractic Literature + PEDro	424	95.9	97.0	100.0	90.6
Cochrane Library + Google Scholar + MEDLINE/PubMed + PEDro	424	95.9	97.0	100.0	90.6
Cochrane Library + Google Scholar + PEDro + AMED	424	95.9	97.0	100.0	90.6
Cochrane Library + Google Scholar + Web of Science + PEDro	424	95.9	97.0	100.0	90.6
CINAHL + Cochrane Library + Google Scholar + AMED	420	95.0	95.6	100.0	88.2
CINAHL + Google Scholar + PEDro + AMED	420	95.0	96.8	100.0	85.9
Cochrane Library + EMBASE + Google Scholar + AMED	420	95.0	95.6	100.0	88.2
Cochrane Library + Google Scholar + Index to Chiropractic Literature + AMED	420	95.0	95.6	100.0	88.2
Cochrane Library + Google Scholar + MEDLINE/PubMed + AMED	420	95.0	95.6	100.0	88.2
Cochrane Library + Google Scholar + Web of Science + AMED	420	95.0	95.6	100.0	88.2
Google Scholar + Index to Chiropractic Literature + PEDro + AMED	420	95.0	96.8	100.0	87.1
CINAHL + EMBASE + Google Scholar + PEDro	419	94.8	96.5	100.0	84.7
CINAHL + Google Scholar + Index to Chiropractic Literature + PEDro	419	94.8	96.5	100.0	84.7
CINAHL + Google Scholar + MEDLINE/PubMed + PEDro	419	94.8	96.5	100.0	84.7
CINAHL + Google Scholar + Web of Science + PEDro	419	94.8	96.5	100.0	84.7
EMBASE + Google Scholar + Index to Chiropractic Literature + PEDro	419	94.8	96.5	100.0	85.9
EMBASE + Google Scholar + PEDro + AMED	419	94.8	96.8	100.0	85.9
Google Scholar + Index to Chiropractic Literature + MEDLINE/PubMed + PEDro	419	94.8	96.5	100.0	85.9
Google Scholar + Index to Chiropractic Literature + Web of Science + PEDro	419	94.8	96.5	100.0	85.9
Google Scholar + MEDLINE/PubMed + PEDro + AMED	419	94.8	96.8	100.0	85.9
Google Scholar + Web of Science + PEDro + AMED	419	94.8	96.8	100.0	85.9
CINAHL + Cochrane Library + EMBASE + Google Scholar	418	94.6	95.3	100.0	84.7
CINAHL + Cochrane Library + Google Scholar + Index to Chiropractic Literature	418	94.6	95.3	100.0	84.7
CINAHL + Cochrane Library + Google Scholar + MEDLINE/PubMed	418	94.6	95.3	100.0	84.7
CINAHL + Cochrane Library + Google Scholar + Web of Science	418	94.6	95.3	100.0	84.7
CINAHL + Cochrane Library + Index to Chiropractic Literature + PEDro	418	94.6	95.6	100.0	87.1
Cochrane Library + EMBASE + Google Scholar + Index to Chiropractic Literature	418	94.6	95.3	100.0	84.7
Cochrane Library + EMBASE + Index to Chiropractic Literature + PEDro	418	94.6	95.6	100.0	87.1
Cochrane Library + Google Scholar + Index to Chiropractic Literature + MEDLINE/PubMed	418	94.6	95.3	100.0	84.7
Cochrane Library + Google Scholar + Index to Chiropractic Literature + Web of Science	418	94.6	95.3	100.0	84.7
Cochrane Library + Index to Chiropractic Literature + MEDLINE/PubMed + PEDro	418	94.6	95.6	100.0	87.1
Cochrane Library + Index to Chiropractic Literature + PEDro + AMED	418	94.6	95.6	100.0	87.1
CINAHL + Cochrane Library + EMBASE + PEDro	417	94.3	95.2	100.0	85.9
CINAHL + Cochrane Library + MEDLINE/PubMed + PEDro	417	94.3	95.2	100.0	85.9
CINAHL + Cochrane Library + Web of Science + PEDro	417	94.3	95.2	100.0	85.9
Cochrane Library + EMBASE + Google Scholar + MEDLINE/PubMed	417	94.3	95.2	100.0	83.5
Cochrane Library + EMBASE + Google Scholar + Web of Science	417	94.3	95.2	100.0	83.5
Cochrane Library + EMBASE + MEDLINE/PubMed + PEDro	417	94.3	95.2	100.0	85.9
Cochrane Library + EMBASE + PEDro + AMED	417	94.3	95.2	100.0	85.9
Cochrane Library + EMBASE + Web of Science + PEDro	417	94.3	95.2	100.0	85.9
Cochrane Library + Google Scholar + MEDLINE/PubMed + Web of Science	417	94.3	95.2	100.0	83.5
Cochrane Library + Index to Chiropractic Literature + Web of Science + PEDro	417	94.3	95.4	100.0	85.9
Cochrane Library + MEDLINE/PubMed + PEDro + AMED	417	94.3	95.2	100.0	85.9
Cochrane Library + MEDLINE/PubMed + Web of Science + PEDro	417	94.3	95.2	100.0	85.9
Cochrane Library + Web of Science + PEDro + AMED	417	94.3	95.2	100.0	85.9
EMBASE + Google Scholar + MEDLINE/PubMed + PEDro	417	94.3	96.4	100.0	84.7
EMBASE + Google Scholar + Web of Science + PEDro	417	94.3	96.4	100.0	84.7
Google Scholar + MEDLINE/PubMed + Web of Science + PEDro	417	94.3	96.4	100.0	84.7
CINAHL + Cochrane Library + PEDro + AMED	416	94.1	95.0	100.0	84.7
CINAHL + EMBASE + Google Scholar + AMED	413	93.4	94.9	100.0	75.3
Cochrane Library + Index to Chiropractic Literature + MEDLINE/PubMed + AMED	413	93.4	93.9	100.0	84.7
CINAHL + Cochrane Library + Index to Chiropractic Literature + AMED	412	93.2	93.8	100.0	84.7
CINAHL + Cochrane Library + MEDLINE/PubMed + AMED	412	93.2	93.5	100.0	83.5
CINAHL + EMBASE + Google Scholar + Index to Chiropractic Literature	412	93.2	94.0	100.0	69.4
CINAHL + EMBASE + Index to Chiropractic Literature + PEDro	412	93.2	95.1	100.0	81.2
CINAHL + EMBASE + PEDro + AMED	412	93.2	95.0	100.0	81.2
CINAHL + Index to Chiropractic Literature + MEDLINE/PubMed + PEDro	412	93.2	95.1	100.0	81.2
CINAHL + MEDLINE/PubMed + PEDro + AMED	412	93.2	95.0	100.0	81.2
Cochrane Library + EMBASE + Index to Chiropractic Literature + AMED	412	93.2	93.8	100.0	84.7
Cochrane Library + EMBASE + MEDLINE/PubMed + AMED	412	93.2	93.5	100.0	83.5
Cochrane Library + Index to Chiropractic Literature + Web of Science + AMED	412	93.2	93.8	100.0	84.7
Cochrane Library + MEDLINE/PubMed + Web of Science + AMED	412	93.2	93.5	100.0	83.5
EMBASE + Google Scholar + Index to Chiropractic Literature + AMED	412	93.2	94.5	100.0	76.5
CINAHL + Cochrane Library + EMBASE + AMED	411	93.0	93.4	100.0	83.5
CINAHL + Cochrane Library + Index to Chiropractic Literature + MEDLINE/PubMed	411	93.0	93.6	100.0	81.2
CINAHL + Cochrane Library + Web of Science + AMED	411	93.0	93.4	100.0	83.5
CINAHL + EMBASE + Web of Science + PEDro	411	93.0	94.7	100.0	80.0
CINAHL + MEDLINE/PubMed + Web of Science + PEDro	411	93.0	94.7	100.0	80.0
Cochrane Library + EMBASE + Index to Chiropractic Literature + MEDLINE/PubMed	411	93.0	93.6	100.0	81.2
Cochrane Library + EMBASE + Web of Science + AMED	411	93.0	93.4	100.0	83.5
Cochrane Library + Index to Chiropractic Literature + MEDLINE/PubMed + Web of Science	411	93.0	93.6	100.0	81.2
EMBASE + Index to Chiropractic Literature + PEDro + AMED	411	93.0	95.0	100.0	80.0
CINAHL + Cochrane Library + EMBASE + Index to Chiropractic Literature	410	92.8	93.4	100.0	81.2
CINAHL + Cochrane Library + EMBASE + MEDLINE/PubMed	410	92.8	93.2	100.0	80.0
CINAHL + Cochrane Library + Index to Chiropractic Literature + Web of Science	410	92.8	93.4	100.0	81.2
CINAHL + Cochrane Library + MEDLINE/PubMed + Web of Science	410	92.8	93.2	100.0	80.0
CINAHL + EMBASE + MEDLINE/PubMed + PEDro	410	92.8	94.6	100.0	78.8
CINAHL + Google Scholar + Index to Chiropractic Literature + AMED	410	92.8	93.2	100.0	65.9
CINAHL + Google Scholar + MEDLINE/PubMed + AMED	410	92.8	93.7	100.0	65.9
CINAHL + Index to Chiropractic Literature + Web of Science + PEDro	410	92.8	94.7	100.0	78.8
Cochrane Library + EMBASE + Index to Chiropractic Literature + Web of Science	410	92.8	93.4	100.0	81.2
EMBASE + Index to Chiropractic Literature + Web of Science + PEDro	410	92.8	94.7	100.0	78.8
Google Scholar + Index to Chiropractic Literature + MEDLINE/PubMed + AMED	410	92.8	94.1	100.0	70.6
Index to Chiropractic Literature + MEDLINE/PubMed + PEDro + AMED	410	92.8	94.9	100.0	78.8
CINAHL + Cochrane Library + EMBASE + Web of Science	409	92.5	93.0	100.0	80.0
CINAHL + Web of Science + PEDro + AMED	409	92.5	94.7	100.0	78.8
Cochrane Library + EMBASE + MEDLINE/PubMed + Web of Science	409	92.5	93.2	100.0	78.8
EMBASE + Google Scholar + Index to Chiropractic Literature + Web of Science	409	92.5	93.3	100.0	67.1
EMBASE + Google Scholar + Web of Science + AMED	409	92.5	93.9	100.0	71.8
EMBASE + Index to Chiropractic Literature + MEDLINE/PubMed + PEDro	409	92.5	94.6	100.0	77.6
Google Scholar + Index to Chiropractic Literature + Web of Science + AMED	409	92.5	94.2	100.0	72.9
Index to Chiropractic Literature + MEDLINE/PubMed + Web of Science + PEDro	409	92.5	94.6	100.0	77.6
CINAHL + EMBASE + Google Scholar + MEDLINE/PubMed	408	92.3	93.3	100.0	67.1
CINAHL + EMBASE + Google Scholar + Web of Science	408	92.3	93.3	100.0	67.1
CINAHL + Google Scholar + Index to Chiropractic Literature + MEDLINE/PubMed	408	92.3	93.2	100.0	64.7
CINAHL + Google Scholar + Web of Science + AMED	408	92.3	93.7	100.0	65.9
EMBASE + Google Scholar + Index to Chiropractic Literature + MEDLINE/PubMed	408	92.3	93.1	100.0	64.7
EMBASE + Google Scholar + MEDLINE/PubMed + AMED	408	92.3	93.7	100.0	70.6
EMBASE + MEDLINE/PubMed + PEDro + AMED	408	92.3	93.9	100.0	76.5
EMBASE + Web of Science + PEDro + AMED	408	92.3	93.9	100.0	76.5
Index to Chiropractic Literature + Web of Science + PEDro + AMED	408	92.3	94.6	100.0	76.5
MEDLINE/PubMed + Web of Science + PEDro + AMED	407	92.1	93.8	100.0	75.3
CINAHL + EMBASE + Index to Chiropractic Literature + AMED	406	91.9	93.4	100.0	77.6
CINAHL + Google Scholar + Index to Chiropractic Literature + Web of Science	406	91.9	93.1	100.0	64.7
EMBASE + MEDLINE/PubMed + Web of Science + PEDro	406	91.9	93.6	100.0	75.3
Google Scholar + MEDLINE/PubMed + Web of Science + AMED	406	91.9	92.8	100.0	62.4
CINAHL + Index to Chiropractic Literature + MEDLINE/PubMed + AMED	405	91.6	93.2	100.0	71.8
Google Scholar + Index to Chiropractic Literature + MEDLINE/PubMed + Web of Science	405	91.6	92.5	100.0	62.4
CINAHL + EMBASE + MEDLINE/PubMed + AMED	404	91.4	92.8	100.0	70.6
CINAHL + Index to Chiropractic Literature + PEDro + AMED	404	91.4	93.0	100.0	71.8
CINAHL + EMBASE + Index to Chiropractic Literature + Web of Science	403	91.2	92.1	100.0	65.9
CINAHL + EMBASE + Web of Science + AMED	403	91.2	92.6	100.0	70.6
CINAHL + Google Scholar + MEDLINE/PubMed + Web of Science	403	91.2	91.9	100.0	60.0
CINAHL + EMBASE + Index to Chiropractic Literature + MEDLINE/PubMed	402	91.0	92.2	100.0	64.7
EMBASE + Index to Chiropractic Literature + MEDLINE/PubMed + AMED	402	91.0	92.3	100.0	69.4
EMBASE + Index to Chiropractic Literature + Web of Science + AMED	402	91.0	92.4	100.0	71.8
EMBASE + Google Scholar + MEDLINE/PubMed + Web of Science	401	90.7	92.2	100.0	62.4
CINAHL + MEDLINE/PubMed + Web of Science + AMED	400	90.5	91.6	100.0	61.2
CINAHL + EMBASE + MEDLINE/PubMed + Web of Science	399	90.3	91.2	100.0	62.4
CINAHL + Index to Chiropractic Literature + MEDLINE/PubMed + Web of Science	399	90.3	91.5	100.0	61.2
EMBASE + Index to Chiropractic Literature + MEDLINE/PubMed + Web of Science	399	90.3	91.1	100.0	60.0
EMBASE + MEDLINE/PubMed + Web of Science + AMED	397	89.8	90.7	100.0	62.4
Index to Chiropractic Literature + MEDLINE/PubMed + Web of Science + AMED	395	89.4	91.6	100.0	61.2
CINAHL + Index to Chiropractic Literature + Web of Science + AMED	387	87.6	90.5	100.0	56.5

^a^Overall recall: The total number of included references retrieved by the database(s) divided by the total number of included references

^b^Mean recall per SR: The average recall rate per SR

^c^Median recall per SR: The median value of recall per SR

^d^100% recall per SR: The percentage of SRs for which the database(s) retrieved all included references

## Abbreviations

SR: Systematic review
RCT: Randomized controlled trial
CENTRAL: The Cochrane Central Register of Controlled Trials
SMT: Spinal manipulative therapy
PRISMA: The preferred reporting items for systematic reviews and meta-analyses
SI: Sacroiliac-joint
PROM: Patient-reported outcome measure
ICL: Index to Chiropractic Literature
DOI: Digital object identifier
IQR: Interquartile range
